# Comparative Effectiveness of Potential Elicitors of Plant Resistance against *Spodoptera frugiperda* (J. E. Smith) (Lepidoptera: Noctuidae) in Four Crop Plants

**DOI:** 10.1371/journal.pone.0136689

**Published:** 2015-09-02

**Authors:** John W. Gordy, B. Rogers Leonard, David Blouin, Jeffrey A. Davis, Michael J. Stout

**Affiliations:** 1 Texas A&M Agrilife Extension, Rosenberg, Texas, United States of America; 2 Louisiana State University Agricultural Center, Baton Rouge, Louisiana, United States of America; 3 Department of Experimental Statistics, Louisiana State University Agricultural Center, Baton Rouge, Louisiana, United States of America; 4 Department of Entomology, Louisiana State University Agricultural Center, Baton Rouge, Louisiana, United States of America; Pennsylvania State University, UNITED STATES

## Abstract

Feeding by insect herbivores activates plant signaling pathways, resulting in the enhanced production of secondary metabolites and other resistance-related traits by injured plants. These traits can reduce insect fitness, deter feeding, and attract beneficial insects. Organic and inorganic chemicals applied as a foliar spray, seed treatment, or soil drench can activate these plant responses. Azelaic acid (AA), benzothiadiazole (BTH), gibberellic acid (GA), harpin, and jasmonic acid (JA) are thought to directly mediate plant responses to pathogens and herbivores or to mimic compounds that do. The effects of these potential elicitors on the induction of plant defenses were determined by measuring the weight gains of fall armyworm, *Spodoptera frugiperda* (J. E. Smith) (FAW) (Lepidoptera: Noctuidae) larvae on four crop plants, cotton, corn, rice, and soybean, treated with the compounds under greenhouse conditions. Treatment with JA consistently reduced growth of FAW reared on treated cotton and soybean. In contrast, FAW fed BTH- and harpin-treated cotton and soybean tissue gained more weight than those fed control leaf tissue, consistent with negative crosstalk between the salicylic acid and JA signaling pathways. No induction or inconsistent induction of resistance was observed in corn and rice. Follow-up experiments showed that the co-application of adjuvants with JA failed to increase the effectiveness of induction by JA and that soybean looper [*Chrysodeixis includens* (Walker)], a relative specialist on legumes, was less affected by JA-induced responses in soybean than was the polyphagous FAW. Overall, the results of these experiments demonstrate that the effectiveness of elicitors as a management tactic will depend strongly on the identities of the crop, the pest, and the elicitor involved.

## Introduction

All plants possess biochemical, morphological, and physiological traits that enable them to reduce the impact of herbivorous arthropods and other biotic threats [1]. Some of these resistance-related traits are expressed by a plant constitutively, regardless of a plant’s history of attack by herbivores. Other resistance-related traits, however, are inducible, meaning they are only expressed, or are expressed to a greater extent, following initial attack by an herbivore or a pathogen [2–4]. Indeed, the ability to respond to herbivory by increasing allocation to resistance is a common, if not universal, attribute of plants [3]. Increases in resistance in plants following herbivory are often plant-systemic and long-lasting, and there is some specificity in both the induction of resistance by different types of herbivory and in the effects of induced resistance on different types of subsequent herbivores [3, 5]. Inducible resistance to herbivores is usually classified as direct (responses that directly and negatively affect the physiology or behavior of herbivores), or indirect (responses that facilitate the activities of natural enemies of herbivores) [1, 5]. Induced resistance is thought to be an adaptation to reduce the costs of expressing resistance-related traits in plants, particularly because threats from herbivores and pathogens can be highly variable in space and time [3].

Rapid, systemic and specific plant responses to herbivore feeding are governed by networks of hormones and other signals [4]. The plant hormones most commonly associated with inducible resistance are jasmonic acid (JA), salicylic acid (SA), and ethylene (ET) [6]. JA is a key regulator of responses to chewing herbivores and necrotrophic pathogens in plants but is also involved in the inhibition of seed germination and plant growth and promotes leaf senescence, fruit abscission, tuber formation, flower and fruit development, pigment formation, and tendril coiling [7]. Levels of endogenous JA increase following attack and, in response, secondary metabolites are produced *in vivo*. These metabolites deter insect feeding, toxify or interfere with acquisition of nutrients by insects, or attract natural enemies [8]. SA, on the other hand, is a key regulator of responses to biotrophic pathogens and piercing-sucking insects [8–10]. Levels of SA increase following attack by biotrophic pathogens and piercing-sucking herbivores, leading to the production of responses associated with resistance to pathogens such as pathogenesis-related proteins [10]. Interactions among these hormones appear to be important, with one of the best-studied interactions being the negative crosstalk that exists among JA- and SA-mediated responses [6]. Other hormones, such as gibberellins and abscisic acid, also play roles in induced resistance, for example as modulators of JA, SA, and ET [3]. Historically, the most important models for the study of the hormonal control of induced responses have been dicots, and it is unclear whether the roles of JA and SA in monocots are identical to their roles in dicots [3, 11].

The increasingly detailed elucidation of the signaling networks that govern inducible resistance has suggested the possibility of using hormones, hormone mimics, or elicitors of hormone-mediated pathways to stimulate plant resistance in a specific and timely manner as a component of integrated management programs [12, 13]. The effects of a large number of potential elicitors on plant resistance to pathogenic microorganisms have been studied [14]. To this point, however, research on elicitors of plant resistance to herbivores has been performed on a fairly restricted set of plants, including tomato, *Arabidopsis*, and cotton [15–19]. Additionally, most individual studies have focused on a single plant species and a limited number of elicitors, notably JA [20–24]. A comprehensive examination of several elicitors across multiple agronomic crops in a single study is lacking in the scientific literature.

The experiments reported here utilized JA and four elicitors previously studied primarily for their effects on disease resistance: azelaic acid (AA), benzothiadiazole (BTH), gibberellic acid (GA), and harpin [22, 23, 25–28], AA is a naturally occurring saturated dicarboxylic fatty acid that has demonstrated anti-inflammatory and antibacterial properties [29], as well as inducing local and systemic resistance to the plant pathogen, *Pseudomonas syringae*, in *Arabidopsis* [27]. BTH is a SA mimic and has shown promise as a systemic inducer of resistance to plant pathogens, but has shown limited effects on reducing damage or on host preference for insects in prior studies [14]. Gibberellins (GA) are terpenoids [30] that are found at highest concentrations in immature seeds and lower concentrations in root and, especially, shoot tissue, comparatively [30]. They are biologically active in plants and cause the elongation of cells, breaking of seed and bud dormancy, and the mobilization of nutrients including the synthesis of hydrolytic enzymes in barley, *Hordeum vulgare* (L.), wheat, *Triticum spp*., and wild oat, *Avena fatua* (L.) [30]. Plant damage from insect feeding can be reduced by GAs [31], and GAs can promote morphological changes resulting in physical defense strategies. When used alone or in conjunction with fenchlorfenuron, a synthetic cytokinin, as a pretreatment for the black pecan aphid, *Melanocallis caryaefoleiae* (Davis) (Hemiptera: Aphididae), on pecan, *Carya illinoensis* (Wangenh), GA significantly reduced leaf chlorosis [31]. Additionally, GA acted synergistically with JA to increase the number and density of leaf trichomes in *Arabidopsis* [22]. Harpin is a protein produced by the bacterium *Erwinia amylovora* [32]. It promotes resistance to plant pathogens including fungi and bacteria [15, 26]. Harpin was shown to induce resistance to the fungal pathogen, *Peronospora parasitica*, and the bacterial pathogen, *Pseudomonas syringae*, in *Arabidopsis* spp., but did not decrease green peach aphid (*Myzus persicae*) populations when applied exogenously to tomato[15, 19].

The primary objective of the current study was to compare the effects of several potential elicitors on the resistance of four crop plants: the monocots maize, *Zea mays* (L.), and rice, *Oryza sativa* (L.), and the dicots cotton, *Gossypium hirsutum* (L.), and soybean, *Glycine max* (L.). The insect species used to assess levels of induced resistance following elicitor application was the fall armyworm (FAW), *Spodoptera frugiperda* (J. E. Smith) (Lepidoptera: Noctuidae). The FAW is a chewing insect that can cause losses in crop yields due to plant defoliation and fruit injury, and is a pest of all four crops used in this study [33–36]. Grasses are preferred by FAW larvae, but the larval stage of FAW is polyphagous and has been observed feeding on the foliage or fruit of over 80 host species of plants, including both monocots and dicots [34]. The FAW is composed of two sympatric strains, morphologically indistinguishable but genetically separable. The “corn strain” is generally associated with corn and other large grasses, whereas the “rice strain” generally associated with rice and pasture grasses [34].

In addition to comparing the effects of the putative elicitors on the resistance of corn, cotton, rice, and soybean to FAW, two follow-up experiments were conducted to test specific hypotheses suggested by the results of the elicitor screening. The first follow-up experiment was conducted in cotton and corn to study the effects of co-application of JA and various adjuvants on FAW resistance. Adjuvants have been used in combination with pesticides as dispersants, stickers, emulsifiers, penetrants, and for other various purposes, since the onset of modern pesticide use [37]. It was hypothesized that addition of adjuvants to solutions of JA would increase the inducing activity of JA by decreasing surface tension of the spray mixture, thus increasing availability of JA for plant uptake. In a second follow-up experiment using soybean, the strength of JA- induced resistance in soybean against FAW, a grass specialist, was compared with the strength of JA-induced resistance against soybean looper (SBL), *Chrysodeixis includens* (Walker) (Lepidoptera: Noctuidae). SBL is a polyphagous defoliating species that has 73 known host-plant species in 29 families [38], including soybean and cotton. However, soybean is the most preferred host for SBL feeding and oviposition [38]. Soybean foliage consumption between R3 (pod initiation) to R6 (full seed) by SBL can cause significant yield loss [39]. This experiment was conducted to test the hypothesis that a relative specialist would be less affected by JA-induced responses than an herbivore with a more generalized host range.

## Methods and Materials

### Plants and insects

Cotton (‘LA1110017’), rice (‘CL131’), and soybean (‘Clifford’) seeds were obtained from breeding programs in the Louisiana State University Agricultural Center. Seeds of field corn (‘Trucker’s Favorite Yellow’, River Valley Heirloom Seeds, Glenwood, AR, USA.) were purchased from a hardware store. All plants were grown in 1.6 liter round (15 cm diameter) plastic pots using commercial potting soil (peat-aged pine bark-perlite, 50-40-10; Sun Gro Horticulture, Bellevue, WA, USA). Plants were maintained in a greenhouse under natural lighting and supplemented with 400 watt metal halide lights placed 1.25 meters above the pots, on a 14:10 h (light: dark) schedule. Growing temperatures ranged from 20°C to 35°C. After plant emergence, granular fertilizer (13.5 g, N-P-K, 13-13-13; Meherrin Fertilizer Inc., Severn, NC, USA) was applied. The plants were watered to maintain adequate soil moisture. Cotton, corn, and rice were grown to the 3–4 leaf stages, and soybean was grown to the V1-V2 stage.

Larval FAW from two separate laboratory colonies were used in these experiments. One colony was initiated using larvae collected from weedy grasses in a fallow field that is rotated into rice every other year at the LSU AgCenter Rice Research Station in Crowley, Louisiana, in 2011. Larvae from this collection were most likely ‘rice-strain’ FAW [34], but this was not confirmed. This strain is referred to below as the “presumptive rice strain”. A second FAW colony was established from larvae collected from a cotton field at the Macon Ridge Research Station in Winnsboro, Louisiana in 2005, and supplemented with larvae collected from field corn in the same area in 2006 and 2008. The FAW populations sampled in Winnsboro have been genetically confirmed as being the corn-strain and will be referred to as such in this paper [40]. The presumptive rice strain and the corn-strain colonies were maintained in separate laboratories on meridic diet. The diets used for rearing of larvae in the two colonies were similar (Fall Armyworm Diet [Southland Products Incorporated, Lake Village, AR, USA] for the presumptive rice strain and Stonefly Heliothis Diet [Ward‘s Natural Science, Rochester, NY] for the corn strain). Pupae were placed in buckets covered with cheese cloth and provided with fresh water and a mixture of honey, beer, water, and ascorbic acid (150ml-150ml-300ml-12g). After emergence, adults mated and females oviposited eggs onto the cheesecloth, which was collected daily and placed in a plastic bag, labeled, and set aside. When neonates began to emerge, they were placed in 8-cell trays (Bio-Serv, Frenchtown, NJ, USA), 20–30 per cell, and supplied with meridic diet. Larvae were kept on artificial diet for approximately six to seven days.

Soybean looper (SBL) larvae were obtained from a colony originating from a collection made in 2008 from a soybean field at the Macon Ridge Research Station near Winnsboro, Louisiana. The colony was maintained in the laboratory following methods described by Brown [41]. The laboratory growth room was kept at approximately 27°C and 80% humidity, under a 14:10 hour light schedule (light:dark). Pupae were placed in covered buckets lined with paper towels and provided with a mixture of 10% v/v honey-water mixture. After emergence, adults mated and females oviposited onto the paper towels, which were collected and placed in a plastic bag, labeled, and set aside. When neonates emerged from eggs, they were placed in one ounce solo cups containing soybean looper diet (Southland Products Incorporated, Lake Village, AR, USA).

### Feeding assays

The effects of elicitor applications on the resistance of plants to FAW were determined by measuring the suitability of foliage from elicitor-treated and untreated plants for larval growth in short-term feeding assays. Feeding assays for all four crop plants were conducted in an identical manner. To initiate assays, six to seven-day old larvae maintained on meridic diet were stage-synchronized by selecting larvae that were about to molt (noticeable gap behind head just prior to slipping head capsule). Synchronized larvae were placed individually into cells of 32-cell trays (Bio-Serv, Frenchtown, NJ, USA) and starved for 18–24 h to ensure that their guts were evacuated before their masses were measured. Larval masses after starvation were determined using a microbalance (model XS105, Mettler-Toledo LLC, Columbus, OH, USA). More larvae were weighed than were needed for each experiment, so that only newly molted larvae with similar masses (mean ± 1 standard deviation) were used in the experiment. Weighed larvae were placed together in 9cm plastic petri dishes (for rice) or 32-cell trays (for cotton, corn, and soybean) containing four layers of cotton batting saturated with deionized water to maintain moisture in excised tissue. Excised leaf tissue from fully emerged true leaves of untreated plants or plants treated 48 hours previously with potential elicitors (see below) were then added to dishes or cells. Larvae were allowed to feed on excised plant tissue for approximately 72 hours and were checked daily to ensure they were not food-limited. After the feeding assay was completed, larvae were returned to individual empty cells for an additional 6–24 h starvation period to ensure that the larval gut was evacuated before final mass was measured and recorded.

### Effects of putative elicitors on plant resistance to fall armyworm

The effects of application of five potential elicitors (BTH, GA, JA, harpin, and AA, Table 1) on the suitability of leaf tissue for FAW growth were compared in a total of 11 experiments, three each with cotton, rice, and soybean, and two with corn. BTH, GA, and harpin were used at rates equivalent to label rates whereas AA and JA were mixed at concentrations of 1.0 and 2.0 mM, respectively (Table 1); these concentrations had been shown to be sufficient to induce responses in prior experiments [31, 32]. The elicitors were thoroughly mixed in 100 ml of deionized water and applied using a gas propellant-powered hand sprayer (Preval, Coal City, IL, USA). The JA was first dissolved in 1 ml of ethanol; the spray jar containing AA and water was placed in a sonicator for approximately five minutes to aid in mixing. The control treatment for all experiments was 1.0% (v/v) ethanol in deionized water. To prevent cross-treatment exposure, each group of plants to be sprayed with a given elicitor was removed from the greenhouse bench, placed in front of an exhaust fan for treatment, and allowed to dry before being returned to common greenhouse area. Separate sprayers were used for each elicitor. None of the elicitor treatments had noticeable phytotoxic effects on plants at the rates used. Plant material for feeding assays (see above) was collected 48 hours after elicitor treatment. Leaf tissue from each treatment was excised from plants using scissors, pooled, transported to the laboratory on ice, and divided evenly among dishes or cells for feeding assays.

**Table 1:** Putative elicitors used in this study.

Chemical	Trade Name (if applicable)	Manufacturer	Rate	Maximum dose per plant*
Azelaic Acid (AA)	n/a	Sigma Aldrich	0.019 g/100ml (1 mM)	1.9 mg
Benzothiadiazole (BTH)	Actigard 50wg	Syngenta Crop Protection	0.005 g/100ml	0.5 mg
Gibberellin (GA_3_)	ProGibb 40%	Valent BioSciences	0.01 g/100ml	1.0 mg
Harpin	Employ H&T	Plant Health Care, Inc.	0.03 g/100ml	3.0 mg
Jasmonic Acid (JA)	n/a	Tokyo Chemical Industry Co., Inc.	0.042 g/100ml (2 mM)	4.2 mg

*Maximum dose per plant was calculated by dividing total amount of elicitor in mixture by the number of plants treated per application (10 for maize, cotton, and soybean). For rice, maximum dose per plant was lower because there were 10–15 plants per pot.

### Replication and statistical analysis

For each of the three experiments with cotton, rice, and soybean, 10 replicates (larvae) were used for each of six treatments (five elicitors and the control), for a total of 170, 176, and 177 observations, respectively (insects that died during feeding assays were excluded). For the two corn experiments, 10 replicates (larvae) were used for each of the six treatments for a total of 114 observations. Presumptive rice strain FAW were used for experiments with rice and corn strain FAW were used for corn, cotton, and soybean. Data from the two experiments with corn or the three experiments with soybean, rice, and cotton were pooled for analysis. For each plant, analysis of covariance (ANCOVA) was performed with final weight as the response variable, initial larva weight as the covariate, treatment (elicitor) as a fixed effect, and experiment as a random effect. The ANCOVA was performed using PROC MIXED in SAS 9.3[42, 43]. Pooled data for each plant species was tested for a treatment by covariate interaction using PROC MIXED. If a significant interaction was found, a contrast was performed comparing treatments at the mean of the covariate. Means were separated using Dunnett’s method for multiple comparisons to a control. Least squares means for estimated final weight are reported in results.

### Effects of adjuvants on the response of corn and cotton to jasmonic acid

Six additional experiments, three with cotton and three with corn, were conducted to test the hypothesis that addition of adjuvants to solutions of JA would increase the effectiveness of JA as an elicitor. Corn and cotton plants were managed as described previously. Corn-strain FAW were used for this experiment and were reared and treated as described previously. Tween 20, Triton X100, Penetrator Plus, and Dyne-Amic (Table 2) were mixed at 50μl per 100ml of solution (0.05% v/v), half the concentration of Tween 20 used in application of JA in previous studies by Bruinsma et al. [44]and Xin et al. [45], and one-fifth the label rate of Penetrator Plus and Dyne-Amic. These rates were selected to minimize phytotoxic effects of JA previously reported [19], while improving penetration and increasing spray coverage [46]. The treatments included: adjuvants with 0.5 mM JA (0.25X rate) (four treatments), adjuvants alone (four treatments), 0.5 mM JA (0.25X rate), 2.0 mM JA (1X rate), and 1.0% ethanol (v/v) in deionized water (control). For cotton and corn, 10 replicates (larvae) were used for each of 11 treatments for a total of 307 and 313 observations, respectively (insects that died during feeding assays were excluded). Elicitor application, feeding assay, and data analyses were performed as described above. Means were separated using Tukey-Kramer’s method for pairwise comparisons.

**Table 2:** Adjuvants Used in this Study.

Chemical type	Trade Name	Manufacturer	Rate	Maximum dose per plant*
Organosilicone surfactant	Dyne-Amic	Helena Chemical Co.	50μl/100ml	5 μl
Nonionic oil surfactant	Penetrator Plus	BASF	50μl/100ml	5 μl
Polyethylene glycol	Triton X100	Sigma Aldrich	50μl/100ml	5 μl
Polyoxyethylene (20) sorbitan monolaurate	Tween 20	Sigma-Aldrich	50μl/100ml	5 μl

*Maximum dose per plant was calculated by dividing total amount of adjuvant in mixture by the number of plants treated per application (10).

### Comparison of jasmonic acid-induced resistance in soybean against fall armyworm and soybean looper

Three additional experiments were conducted with both SBL and FAW to compare the strength of induced resistance in soybean against FAW and SBL. Leaf tissue used to feed both SBL and FAW were from the same sets of treated and untreated plants, and feeding assays with the two species were run simultaneously. For each experiment and species combination, ten replicates (larvae) were used for each of the two treatments. Plant growth, JA treatment (2 mM), and feeding assays followed the methods described above. Data from all three experiments were combined for separate statistical analyses for SBL and FAW, with a total of 88 and 81 observations, respectively (insects that died during feeding assays were excluded). For SBL experiments one and two, third instars were used; for experiment three, second instars were used. Statistical analyses were performed as previously described, with statistical comparisons made only within the same insect species.

## Results

### Effects of putative elicitors on induced plant resistance to fall armyworm

For all four crop plants, there was a significant initial weight (covariate) effect (*F*_1,106_ = 5.94, *P* = 0.0165, *F*_1,161_ = 16.38, *P* < 0.001, *F*_1,167_ = 139.98, *P* < 0.001, and *F*_1,168_ = 41.45, *P* < 0.001, for corn, cotton, rice, and soybean, respectively). For cotton, elicitor treatment had a significant effect on final weight of FAW larvae (*F*_5,161_ = 19.24, *P* < 0.001) (Table 3). Weight gain of larvae fed BTH- or harpin-treated cotton was greater than larvae fed non-treated cotton (*P* = 0.0071 and *P* = 0.0141, respectively). Final weights of larvae reared on JA-treated cotton were lower than larvae reared on non-treated cotton (*P* < 0.001). For soybean, elicitor treatment had a significant effect on final weight of FAW larvae (*F*_5,168_ = 12.81, *P* < 0.001). Weight gain of larvae on harpin-treated soybean foliage was greater than weight gain of larvae on non-treated soybean (*P* = 0.0313). Weight gain of larvae on JA-treated soybean was lower than larvae offered non-treated soybean (*P* < 0.001). For corn and rice, there were no significant treatment effects (*F*_5,106_ = 1.03, *P* = 0.4039 and *F*_5,167_ = 1.15, *P* = 0.3349, respectively).

**Table 3:** Least Squares Means Estimates^1^ for Final Larval Weights (mg ± S.E.) of Fall Armyworm Larvae Reared on Elicitor-Treated Plant Material.

	Crop Plant^1^			
Treatment	Corn^2^ ^,^ ^3^	Cotton^2^ ^,^ ^3^	Rice^2^ ^,^ ^4^	Soybean^2^ ^,^ ^3^
Control	70.5 ± 9.6 a	28.4 ± 6.3 b	52.4 ± 10.9 a	40.6 ± 21.6 b
Azelaic Acid	70.5 ± 9.6 a	34.3 ± 6.3 ab	50.4 ± 10.9 a	44.0 ± 21.6 ab
Benzothiadiazole	70.1 ± 9.6 a	36.0 ± 6.3 a	48.0 ± 10.8 a	41.2 ± 21.6 ab
Gibberellic Acid	60.9 ± 9.9 a	30.3 ± 6.3 ab	49.6 ± 10.8 a	36.8 ± 21.6 ab
Harpin	64.7 ± 9.6 a	35.5 ± 6.3 a	54.1 ± 10.8 a	48.8 ± 21.6 a
Jasmonic Acid	64.7 ± 9.6 a	14.7 ± 6.4 c	47.4 ± 10.9 a	26.3 ± 21.6 c
Treatment p-value	ns	**	ns	**
Initial weight p-value	*	**	**	**

^1^ Estimate based on LS means from analysis of covariance performed in SAS 9.3.

^2^ Numbers in the same column followed by same letter are not significantly different (P < 0.05) as determined by Dunnett’s method for multiple comparisons to a control.

^3^ For corn, cotton, and soybean, corn-strain FAW were used in feeding assays.

^4^ For rice, presumptive rice-strain FAW were used in feeding assays

*indicates *P*<0.05

**indicates *P*<0.001.

### Effects of adjuvants on jasmonic acid-induced resistance to fall armyworm

For both corn and cotton, there was a significant initial weight (covariate) effect (*F*_1,293_ = 165.14, *P* < 0.001 and *F*_1,299_ = 125.41, *P* < 0.001, respectively) (Table 4). For corn, treatment had a significant effect on weight gain of FAW (*F*_10,299_ = 3.89, *P* < 0.001). With the exception of Tween 20 and Penetrator Plus, all treatments resulted in reduced weight gain of FAW compared to the control-treated corn. For cotton, elicitor treatment had a significant effect on the growth of FAW (*F*_10,293_ = 11.15, *P* < 0.001). Larvae of FAW fed cotton treated with 2.0 mM JA gained less weight than all adjuvant treatments (*P* < 0.001), and weight gains of larvae in the 2.0 mM JA treatment were lower than weight gains of larvae fed untreated cotton (*P* < 0.001).

**Table 4:** Least Squares Means Estimates^1^ for Final Larval Weights (mg ± S.E.) of Fall Armyworms Reared on Treated Plant Material.

	Crop Plant^1^	
Treatment	Corn^2^ ^,^ ^3^	Cotton^2^ ^,^ ^3^
Control	126.4 ± 14.7 a	34.1 ± 6.1 abcd
Triton X100	106.3 ± 14.7 b	38.8 ± 6.1 ab
Tween 20	113.0 ± 14.7 ab	40.8 ± 6.1 a
Penetrator Plus	107.9 ± 14.8 ab	41.6 ± 6.1 a
Dyne-Amic	107.3 ± 14.7 b	35.0 ± 6.1 abc
0.5 mM JA	101.5 ± 14.7 b	30.7 ± 6.1 bcd
2.0 mM JA	106.5 ± 14.7 b	21.7 ± 6.1 e
0.5 mM JA + Triton X100	104.7 ± 14.7 b	31.0 ± 6.1 bcd
0.5 mM JA + Tween 20	103.7 ± 14.7 b	28.5 ± 6.1 cde
0.5 mM JA + Penetrator Plus	97.0 ± 14.7 b	29.2 ± 6.1 cde
0.5 mM JA + Dyne-Amic	101.0 ± 14.7 b	26.4 ± 6.1 de
Treatment p-value	*	*
Initial weight p-value	*	*

^1^ Estimate based on LS means from analysis of covariance performed in SAS 9.3.

^2^ Numbers in the same column followed by same letter are not significantly different (P < 0.05) as determined by Tukey-Kramer’s method for pairwise comparisons.

^3^ Corn-strain FAW were used in all assays.

*indicates P<0.001.

### Comparison of jasmonic acid-induced resistance in soybean against fall armyworm and soybean looper

Application of JA to soybean plants reduced the growth of both FAW and SBL (*F*_1,78_ = 37.46, *P <*0.001 and *F*_1,85_ = 9.59, *P* = 0.0026, respectively) (Table 5). However, the resistance induced in soybean by JA was much stronger against FAW (~38% reduction in growth) than against SBL (~9% reduction in growth). There was a significant initial weight (covariate) effect for SBL (*F*_1,85_ = 339.31, *P* < 0.001), but not for FAW (*F*_1,78_ = 0.02, *P- =* 0.8791).

**Table 5:** Least Squares Means Estimates^1^ for Final Larval Weights (mg ± S.E.) of Soybean Loopers and Fall Armyworms Reared on Treated Plant Material.

	Insect ^1^	
Treatment	SBL	FAW
Control	184.5 ± 3.5	52.7 ± 2.3
2.0 mM JA	169.27 ± 14.7	32.6 ± 2.4
Treatment p-value	*	**
Initial weight p-value	**	NS

^1^ Estimate based on LS means from analysis of covariance performed in SAS 9.3.

*indicates P<0.05

**indicates P<0.001.

## Discussion

The primary objective of this study was to screen potential elicitors for their use in inducing resistance to herbivorous insects of major agricultural commodities. Application of JA reduced the suitability of leaves as food for herbivores, as evidenced by reduced growth of FAW larvae in short-term feeding assays on JA-treated compared to control leaf tissue. Similar stimulation of resistance by exogenous JA has been found in numerous prior studies [16, 19, 32, 47]. The reductions in FAW growth found in this study ranged from less than 10% (non-significant) to almost 50%. Importantly, responses to JA were stronger and more consistent in the dicotyledonous crops, cotton and soybean, than in the monocotyledonous crops, corn and rice. In the first set of experiments (screening of elicitors), statistically significant reductions in FAW growth were found only in treated cotton and soybean and not in treated corn and rice. In the second set of experiments (effects of adjuvants), exogenous JA resulted in increased FAW resistance in both corn and cotton, although again the response to 2.0 mM JA was greater in cotton than in corn. The magnitude of JA induction in dicots in these experiments was similar to that observed in previous experiments. For example, weight gain of the generalist *Helicoverpa zea* (Boddie) was reduced by 55–75% on four genetic variants of tomato treated with 2.5 mM methyl jasmonate[48].

There are several possible explanations for the stronger responses of cotton and soybean to JA and the failure to see consistent reductions in FAW growth on JA-treated corn and rice. First, differences among these plant species in their responsiveness to JA may have occurred because JA did not penetrate the cuticles of corn and rice due to inherent differences in plant cuticle between the selected monocots and dicots. In support of this hypothesis (reduced cuticular penetration in monocots), spray mixtures were observed to adhere better to cotton and soybean foliage than corn and rice foliage, spreading better and showing less run-off, whereas, on corn and rice, mixtures appeared to simply form beads and roll off the plant (JWG, personal observations). There is considerable variation in structure and permeability of plant cuticles, which could account for the observed differences in water adherence in monocots and dicots in this study [49]. As a result of decreased adherence, less elicitor might be available for plant uptake in corn and rice, thus reducing activation of defense responses. To test the hypothesis of reduced cuticle penetration in the monocots, a set of experiments using several adjuvants was conducted to investigate the possibility that the lack of JA effects on growth of FAW on corn and rice compared to cotton and soybean was the result of decreased adherence of spray mixtures to corn and cotton. The addition of an adjuvant to 0.5 mM JA did not result in greater reductions in FAW growth than 0.5 mM JA alone for either corn or cotton. Interestingly, however, application of the adjuvants Triton X-100 and Dyne-Amic alone reduced growth of larvae on corn leaves, indicating that these adjuvants either have direct negative effects on larvae or injure plants in ways that induce JA-related or other responses in plants. The lack of differences in growth of larvae fed control and adjuvant-treated cotton leaves, however, argues against the hypothesis that adjuvants directly affect FAW larvae via anti-feedant or toxic effects. Overall, the adjuvant experiments provided little support for the idea that the low responsiveness of corn and rice to JA in the first set of experiments was due to the inability of JA to penetrate the plant cuticle, and point to other explanations for the low effectiveness of JA in corn and rice.

A second hypothesis to account for differences in the effectiveness of JA among the four plants tested is that FAW larvae may be more tolerant of the resistance-related responses elicited by JA in monocots than in dicots. Although the FAW is a polyphagous species, it is a specialist on grasses, and thus might be expected to be relatively tolerant of any changes in biochemistry (direct defenses) induced in corn and rice. This hypothesis was supported by two pieces of evidence from these experiments. First, responsiveness to JA seemed to be inversely correlated with suitability of untreated leaves for growth of larvae (i.e., induction of resistance by JA was stronger in cotton and soybean, plants on which growth of larvae was lowest) (Table 3). Second, in the third set of experiments reported here (comparison of induced resistance in soybean to SBL and FAW), SBL, which specializes on legumes, was less affected by JA-induced responses in soybean than was the generalist FAW, consistent with the general hypothesis that specialists are less affected than generalists by induced changes in the host plants to which they are adapted [50]. Alternatively, different plant species employ different defense strategies, and corn, rice, and cotton have been shown to produce volatiles that may act as attractants to parasitoids and other natural enemies [1, 51–54]. It is possible that the primary defense response in corn and rice is an indirect defense mechanism to attract natural enemies of pests, which these experiments were not designed to test, as opposed to a direct induced chemical defense mechanism.

As an extension of the hypothesis discussed in the previous paragraph, the use of FAW from two separate colonies may have contributed to some of the differences in results among experiments with different host plants. Experiments with corn, cotton and soybean utilized corn strain FAW, whereas experiments with rice utilized FAW from the presumptive rice strain., It is possible that FAW from the presumptive rice strain colony were not as sensitive to JA-induced changes in host plants as FAW from the corn strain. This explanation seems unlikely, however, as the rice strain has not been found to be any less sensitive to differences in host plant than the corn strain in prior experiments [26].

A third hypothesis to account for differences among the four plant species in the effectiveness of JA as an inducer is that the monocots and dicots used in this study differed in their inherent sensitivity to exogenous JA. This is plausible, as a few studies suggest SA and JA signaling in monocots and dicots may differ in subtle ways [11], but further investigations that incorporate quantification of changes in gene and metabolite expression following JA treatment will be needed to characterize possible differences in responsiveness of monocots and dicots to JA.

Some inconsistencies were observed among the results of the elicitor screening experiments and the adjuvant experiments. Most notably, applications of 0.5 mM and 2.0 mM JA alone to corn reduced growth of FAW by 16–20% in the adjuvant experiments, but application of 2 mM JA alone did not cause significant reductions in FAW growth in the initial screening of elicitors (although, numerically, growth was reduced by 8% on JA-treated corn leaves relative to control leaves in the screening experiments). The superior overall growth of FAW larvae in the adjuvant experiments compared to the screening experiments may have allowed a more sensitive test of the effects of JA on corn suitability for FAW; alternatively, differences in the levels of statistical significance in the results of the two sets of experiments may have been an artifact of differences in the means separation tests used (Dunnett versus Tukey). The findings in this second set of experiments indicate that field corn can respond to exogenous application of JA, and that FAW is susceptible to direct induced defenses of corn elicited by JA. In the adjuvant experiments, the application of 2.0 mM JA but not 0.5 mM JA to cotton resulted in reduced weight gain compared to the control-treated plants, suggesting a dose-dependence in responsiveness to JA.

Of the five putative elicitors, JA was the only one to consistently reduce weight gains of FAW when larvae consumed treated leaf tissue, especially from cotton and soybean. In contrast to the effects of JA, FAW fed on leaf material treated with AA, BTH, and harpin demonstrated greater larval weights compared to the control, although the increases were not always significant. These elicitors have previously been shown to promote resistance to plant pathogens, probably by activating the SA-mediated pathway [15, 26, 31, 55]. Increased weight gain of FAW larvae fed leaves treated with these specific elicitors is consistent with other research showing crosstalk between the SA and JA signaling pathways [56]. The activation of the SA pathway, responsible for resistance to some pathogens and piercing-sucking insects, can have a suppressive effect on the JA pathway and induced defenses, especially against chewing insect herbivores [6, 47, 56, 57].

The results presented here agree with those in previous studies that demonstrate induction of plant defenses by JA [16, 18, 32, 47] but not SA or its mimics [20, 58]. The results with harpin are supported by Boughton et al.[18], who showed that harpin was not effective in reducing growth of insect populations on treated plants. Our findings indicated no significant negative effect of BTH-treated tissue on FAW weight gain. These results are similar those from Bi et al. [20]and Inbar et al. [58]which found that BTH- treated cotton had no effect on growth of corn earworm, *H*. *zea*, or cotton bollworm, *Helicoverpa armigera* (Hübner). However, Boughton et al. [18] demonstrated that BTH did decrease development of green peach aphid populations on tomato. The SA pathway is believed to be more responsive to and instrumental in plant defense against piercing-sucking arthropods and biotrophic pathogens [8–10, 59], and can have a suppressive effect on the JA pathway. The findings presented here, in conjunction with prior literature, further support the suggested roles of SA and JA in defense signaling in piercing-sucking versus chewing insect herbivores.

Integrated pest management (IPM) is a multifaceted approach to mitigate damage by herbivorous insect pests which unfortunately still relies too heavily on broad-spectrum synthetic insecticides for many crop-pest systems. The use of elicitors for the induction of plant defenses that result in decreased herbivore fitness may be an additional tactic in IPM programs. The results of these experiments, although they were conducted with only a single variety of each crop and thus must be interpreted cautiously (because induced responses are known to differ among genotypes of a single plant species), nonetheless add to the large number of studies that support the potential use of elicitors in pest management [12–14]. More importantly, the results of these studies suggest several areas in which more research is needed before integration of elicitors into IPM programs can occur. First, these results indicate that more information will be needed on inherent differences in JA and SA signaling among monocots and dicots before comprehensive strategies for using elicitors in pest management can be developed. Second, although use of adjuvants failed to increase induction in these experiments, methods for increasing penetration, uptake, and effectiveness of elicitors will probably be needed to make the tactic cost-effective. Third, the effectiveness of an elicitor as an IPM tactic clearly will also depend on the sensitivities of target pests to the elicitor-induced changes in plants. Finally, although the results reported here indicated that 48 h is sufficient time for some plants to initiate defensive responses following elicitor treatment, more detailed information will be needed on the rapidity and duration of induced resistance following elicitation. Overall, the results of these experiments demonstrate that the effectiveness of elicitors as a management tactic will depend strongly on the identities of the crop, the pest, and the elicitor involved.

## Supporting Information

S1 Dataset MS Word file containing raw data with SAS code used for analyses. (DOC)

## References

[1] Schoonhoven LM, Van Loon JJA, Dicke M. Insect Plant Biology. New York: Oxford University Press; 2005.

[2] Jones JDG, Dangl JL. The plant immune system. Nature 2006; 444: 323–329. 17108957 10.1038/nature05286

[3] Karban R. The ecology and evolution of induced resistance against herbivores. Function. Ecol. 2011; 25: 339–347.

[4] Campos ML, Kang J-H, Howe GA. Jasmonate-triggered plant immunity. J.Chem. Ecol. 2014; 40, 657–675. 10.1007/s10886-014-0468-3 24973116 PMC4143990

[5] Stout MJ. Types and mechanisms of rapidly-induced resistance to herbivorous arthropods In: Walters D, Newton A, Lyon G, editors. Induced resistance for plant defence: A sustainable approach to crop protection, 2nd edition New York: John Wiley and Sons 2014 Pp. 81–105.

[6] Pieterse CMJ, Zamioudis C, Van der Does D, Van Wees SCM. 2014 Signalling networks involved in induced resistance In: Walters D, Newton A, Lyon G, editors. Induced resistance for plant defence: A sustainable approach to crop protection, 2nd edition New York: John Wiley and Sons Pp. 58–80.

[7] Davies PJ. The plant hormones: Their nature, occurrence, and functions In: Davies PJ, editor Plant Hormones: physiology, biochemistry, and molecular biology. The Netherlands: Kluwer Academic Publishers;1995 pp. 1–13.

[8] Smith JL, De Moraes CM, Mescher MC. Jamonate- and salicylate-mediated plant defense responses to insect herbivores, pathogens and parasitic plants. Pest Manag. Sci. 2009; 65: 497–503. 10.1002/ps.1714 19206090

[9] Glazebrook J. Contrasting mechanisms of defense against biotrophic and necrotrophic pathogens. Annu. Rev. Phytopathol. 2005; 43: 205–227. 16078883 10.1146/annurev.phyto.43.040204.135923

[10] Goggin FL. Plant-aphid interactions: molecular and ecological perspectives. Curr. Op. Plant Biol. 2007; 10: 399–408. 10.1016/j.pbi.2007.06.004 17652010

[11] Tamaokia D, Shigemi S, Yamada S, Akihito K, Ayumi M, Hodaka S, et al Jasmonic acid and salicylic acid activate a common defense system in rice. Plant Signal. Behav. 2013; 10.4161/psb.24260 PMC3906320 23518581

[12] Stout MJ, Zehnder GW, Baur ME. Potential for the use of elicitors of plant resistance in arthropod management programs. Arch. Ins. Biochem. Physiol. 2002; 51: 222–235. 10.1002/arch.10066 12432521

[13] Reglinski T, Dann E, Deverall B. Implementation of induced resistance for crop protection In: Walters D, Newton A, Lyon G, editors. Induced resistance for plant defence: A sustainable approach to crop protection, 2nd edition New York: John Wiley and Sons 2014 Pp. 249–299.

[14] Lyon GD. Agents that can elicit induced resistance In: Walters D, Newton A, Lyon G, editors. Induced resistance for plant defence: A sustainable approach to crop protection, 2nd edition New York: John Wiley and Sons 2014 Pp. 11–40.

[15] Dong H, Delaney TP, Bauer DW, Beer SV. Harpin induces disease resistance in Arabidopsis through the systemic acquired resistance pathway mediated by salicylic acid and the NIM1 gene. Plant J. 1999; 20: 207–215. 10571880 10.1046/j.1365-313x.1999.00595.x

[16] Omer AD, Granett J, Karban R, Villa EM. Chemically-induced resistance against multiple pests in cotton. Intl. J. Pest Manag. 2001; 47: 49–54.

[17] Thaler JS, Stout MJ, Karban R, Duffey SS. Jasmonate-mediated induced plant resistance affects a community of herbivores. Ecol. Entomol. 2001; 26: 312–324.

[18] Boughton AJ, Felton GW, Hoover K. Methyl jasmonate application induces increased densities of glandular trichomes on tomato, Lycopersicon esculentum. J. Chem. Ecol. 2005; 31: 2211–2216. 16132222 10.1007/s10886-005-6228-7

[19] Boughton AJ, Hoover K, Felton GW. Impact of chemical elicitor applications on greenhouse tomato plants and population growth on the green peach aphid, Myzus persicae. Ent. Exp. et Applic. 2006; 120: 175–188.

[20] Bi JL, Murphy JB, Felton GW. Does salicylic acid act as a signal in cotton for induced resistance to Helicoverpa zea? J. Chem. Ecol. 1997; 23: 1805–1818.

[21] Black CA, Karban R, Godfrey LD, Grannett J, Chaney WE. Jasmonic acid: a vaccine against leafminers (Diptera: Agromyzidae) in celery. Environ. Entomol. 2003; 32: 1196–1202.

[22] Traw BM, Bergelson J. Interactive effects of jasmonic acid, salicylic acid, and gibberellin on induction of trichomes in Arabidopsis. Plant Physiol. 2003; 133: 1367–1375. 14551332 10.1104/pp.103.027086 PMC281631

[23] Nombela G, Pascual S, Aviles M, Guillard E, Muniz M. Benzothiadiazole induces local resistance to Bemisia tabaci (Hemiptera: Aleyrodidae) in tomato plants. J. Econ. Entomol. 2005; 98: 2266–2271. 16539159 10.1093/jee/98.6.2266

[24] Weston PA. Plant defense elicitors fail to protect Viburnum dentatum from herbivory by viburnum leaf beetle (Coleoptera: Chrysomalidae). J. Econ. Entomol. 2008; 101: 1466–1470. 18767761 10.1603/0022-0493(2008)101[1466:pdeftp]2.0.co;2

[25] Kahl J, Siemens DH, Aerts RJ, Gäbler R, Kühnemann F, Preston CA, et al Herbivore-induced ethylene suppresses a direct defense but not a putative indirect defense against an adapted herbivore. Planta 2000; 210: 336–342. 10664141 10.1007/PL00008142

[26] Yang B, Shiping T, Jie S, Yonghong G. Harpin induces local and systemic resistance against *Trichothecium roseum* in harvested hami melons. Postharvest Biol. Technol. 2005; 38: 183–187.

[27] Jung HW, Tschaplinski TJ, Wang L, Glazebrook J, Greenberg JT. Priming in systemic plant immunity. Science 2009; 324: 89–91. 10.1126/science.1170025 19342588

[28] Hamm JC, Stout MJ, Riggio RM. Herbivore- and elicitor-induced resistance in rice to the rice water weevil (Lissorhoptrus oryzophilus Kuschel) in the laboratory and field. J. Chem. Ecol. 2010; 36: 192–199. 10.1007/s10886-010-9751-0 20151182

[29] Garelnabi M, Litvinov D, Parthasarathy S. Evaluation of a gas chromatography method for azelaic acid determination in selected biological samples. N. Am. J. Med. Sci. 2010; 2: 397–402. 10.4297/najms.2010.2397 22558586 PMC3339096

[30] Naqvi SSM. Plant growth hormones: growth promoters and inhibitors In: Pessarakli M, editor, Handbook of plant and crop physiology. New York: Marcel Dekker New York; 2001, pp. 501–525.

[31] Cottrell TE, Wood BW, Ni X. Application of plant growth regulators mitigates chlorotic foliar injury by the black pecan aphid (Hemiptera: Aphidae). Pest Manag. Sci. 2010; 66: 1236–1242. 10.1002/ps.2000 20715019

[32] Baker CJ, Orlandi EW, Mock NM. Harpin, an elicitor of the hypersensitive response in tobacco caused by *Erwinia amylovora*, elicits active oxygen production in suspension cells. Plant Physiol. 1993; 102: 1341–1344. 12231911 10.1104/pp.102.4.1341 PMC158926

[33] Luttrell RG, Mink JS. Damage to cotton fruiting structures by the fall armyworm, *Spodoptera frugiperda* (Lepidoptera: Noctuidae). J. Cotton Sci. 1999; 3: 35–44.

[34] Meagher RL, Nagoshi RN. Differential feeding of fall armyworm (Lepidoptera: Noctuidae) host strains on meridic and natural diets. Ann. Entomol. Soc. Am. 2012; 105: 462–470.

[35] Musser FR, Catchot AL Jr, Davis JA, Herbert DA Jr, Leonard BR, Lorenz GM, et al 2011 Soybean insect losses in the Southern US. Midsouth Entomol. 2012; 5: 11–17.

[36] Williams MR. Cotton insect losses 2012; 2013. Available: http://www.biochemistry.msstate.edu/resources/croplosses/2012loss.asp. Accessed 2013 Mar 5.

[37] Stevens PJG. Organosilicone surfactants as adjuvants for agrochemicals. Pestic. Sci. 1993; 38: 103–122.

[38] Herzog DC. Sampling soybean looper in soybean In Kogan M, Herzog DC, editors. Sampling methods in soybean entomology. New York: Springer-Verlag;1980 Pp. 141–168.

[39] Turnipseed SG, Kogan M. Integrated control of insects In: Wilcox JR, editor. Soybeans: Improvement, Production, and Uses. Madison, Wisconsin: American Society of Agronomy Wisconsin; 1987 pp. 779–817.

[40] Hardke JT. Contribution of Bacillus Thuringiensis cotton cultivars and insecticides to a fall armyworm, Spodoptera frugiperda, (J.E. Smith), (Lepidoptera: Noctuidae) management strategy. M.Sc. Thesis, Louisiana State University. Electronic Dissertation. 2011. Available: http://etd.lsu.edu/docs/available/etd-07012011-141828/

[41] Brown SA. Evaluating the efficacy of methoxyfenozide on Louisiana, Texas, and the Mid-Southern soybean looper populations. M.Sc. Thesis, Louisiana State University. 2012. Available: http://etd.lsu.edu/docs/available/etd-04202012-150457/

[42] SAS Institute. 2010.

[43] Stout MJ, Riggio MR, Yang Y. Direct induced resistance in Oryza sativa to Spodoptera frugiperda. Environ. Entomol. 2009; 38: 1174–1181. 19689897 10.1603/022.038.0426

[44] Bruinsma M, Van Dam NM, Van Loon JJA, Dicke M. Jasmonic acid-induced changes in Brassica oleracea affect oviposition of two specialist herbivores. J. Chem. Ecol. 2007; 33: 655–668. 17334923 10.1007/s10886-006-9245-2 PMC1915630

[45] Xin Z, Yu Z, Erb M, Turlings TCJ, Wang B, Qi J, et al The broad-leaf herbicide 2,4-dichlorophenoxyacetic acid turns rice into a living trap for a major insect pest and a parasitic wasp. New Phytol. 2012; 194: 498–51. 10.1111/j.1469-8137.2012.04057.x 22313362

[46] Singh M, Mack RE. Effect of organosilicone-based adjuvants on herbicide efficacy. Pestic. Sci. 1993; 38: 219–225.

[47] Thaler JS, Fidantsef AL, Duffey SS, Bostock RM. Trade-offs in plant defense against pathogens and herbivores: a field demonstration of chemical elicitors of induced resistance. J. Chem. Ecol. 1999; 25: 1597–1609.

[48] Tian D, Peiffer M, De Moraes CM, Felton GW. Roles of ethylene and jasmonic acid in systemic induced defense in tomato (Solanum lycoperiscum) against Helicoverpa zea. Planta 2014; 239: 577–589. 10.1007/s00425-013-1997-7 24271004

[49] Schönherr J, Baur P. Modelling penetration of plant cuticles by crop protection agents and effects of adjuvants on their rates of penetration. Pestic. Sci. 1994; 42: 185–208.

[50] Ali JC, Agrawal AA. Specialist versus generalist insect herbivores and plant defense. Trends Plant Sci. 2012; 17: 293–302. 10.1016/j.tplants.2012.02.006 22425020

[51] Rodriguez-Saona C, Crafts-Brandner SJ, Cañas LA. Volatile emissions triggered by multiple herbivore damage: beet armyworm and whitefly on cotton plants. J. Chem. Ecol. 2003; 29: 2539–2550. 14682532 10.1023/a:1026314102866

[52] Rostás M, Turlings TCJ. Induction of systemic acquired resistance in Zea mays also enhances the plant’s attractiveness to parasitoids. Biol. Cont. 2008; 46: 178–186.

[53] Yuan JS, Köllner TG, Wiggins G, Grant J, Degenhardt J, Chen F. Molecular and genomic basis of volatile-mediated indirect defense against insects in rice. Plant J. 2008; 55: 491–503. 10.1111/j.1365-313X.2008.03524.x 18433439

[54] Köllner TG, Gershenzon J, Degenhardt J. Molecular and biochemical evolution of maize terpene synthase 10, an enzyme of indirect defense. Phytochem. 2009; 70: 1139–1145. 10.1016/j.phytochem.2009.06.011 19646721

[55] Barilli E, Prats E, Rubiales E. Benzothiadiazole and BABA improve resistance to Uromyces pisi (Pers.) Wint. in *Pisum sativum* L. with an enhancement of enzymatic activities and total phenolic content. Eur. J. Plant Pathol. 2010; 128: 483–493.

[56] Thaler JS, Agrawal AA, Halitschke R. Salicyate-mediated interactions between pathogens and behaviors. Ecology 2010; 91: 1075–1082. 20462121 10.1890/08-2347.1

[57] Felton GW, Korth KL. Trade-offs between pathogen and herbivore resistance. Curr. Op. Plant Biol. 2000; 3: 309–314. 10.1016/s1369-5266(00)00086-8 10873851

[58] Inbar M, Doostdar H, Gerling D, Mayer RT. Induction of systemic acquired resistance in cotton by BTH has a negligible effect on phytophagous insects. Ent. Exp. Applic. 2001; 99: 65–70.

[59] Leitner M, Boland W, Mithöfer A. 2005. Direct and indirect defenses induced by piercing-sucking and chewing herbivores in *Medicago truncatula* . New Phytol. 2005; 167: 597–606. 15998409 10.1111/j.1469-8137.2005.01426.x

