# Supplementary material for: Comparative Effectiveness of Potential Elicitors of Plant Resistance against Spodoptera frugiperda (J. E. Smith) (Lepidoptera: Noctuidae) in Four Crop Plants
Source: PLoS One. 2015 Sep 2;10(9):e0136689. doi: 10.1371/journal.pone.0136689 (PMC4557932; doi:10.1371/journal.pone.0136689)
Supplement: S1 Dataset — (DOC) [file pone.0136689.s001.doc]

Corn – Initial

dm 'log;clear;output;clear';

options nodate nocenter pageno=1 ls=78 ps=55;

title1 "Corn Combined";

data one;

input InsectID $ IBW FBW TRT $ Block $;

datalines;

2 4.9 57.4 AC 713

4 4.5 95.1 AC 713

12 5.3 93.6 AC 713

14 4.4 61.4 AC 713

16 4.7 48.8 AC 713

19 4.3 49.3 AC 713

28 4.5 85.8 AC 713

29 5.3 69 AC 713

30 5.2 100.5 AC 713

47 4.3 47.5 AC 713

52 4.3 76.8 G 713

58 4.3 55 G 713

63 4.8 51.4 G 713

66 4.4 63.3 G 713

67 5.3 69 G 713

75 4.8 73.8 G 713

79 4.5 88.6 G 713

81 4.3 54.7 G 713

85 4.6 98.2 G 713

90 5 72.4 G 713

101 4.3 61.2 B 713

103 4.7 82.2 B 713

104 5 71.7 B 713

114 4.9 74 B 713

116 4.5 40.2 B 713

119 5 77 B 713

120 4.4 82.1 B 713

121 5 106 B 713

125 4.9 101.3 B 713

129 4.8 64.6 B 713

130 5.2 84.8 H 713

132 4.8 65.1 H 713

138 5.1 113.6 H 713

139 4.4 99.7 H 713

146 5.3 69.3 H 713

151 4.4 13.8 H 713

153 4.4 53.4 H 713

154 5 64.7 H 713

160 4.9 72.5 H 713

161 4.6 52.4 H 713

166 5.3 72 J 713

167 4.4 62.5 J 713

170 4.8 63.9 J 713

175 4.7 84.8 J 713

178 5.2 69.7 J 713

181 4.3 52.6 J 713

182 4.6 101.3 J 713

186 4.7 73 J 713

191 4.7 50.4 J 713

198 4.8 94.1 J 713

211 5.3 116.6 AZ 713

213 5.1 86.7 AZ 713

218 4.6 77.1 AZ 713

221 4.5 69.5 AZ 713

225 4.3 49.9 AZ 713

228 4.9 52.2 AZ 713

240 4.7 65.1 AZ 713

243 5.2 64.9 AZ 713

249 4.5 64.3 AZ 713

250 5.2 58.5 AZ 713

1 6 68.2 AC 720

2 5.1 55.1 AC 720

4 5.2 70.6 AC 720

7 6.3 96 AC 720

8 5.6 62.8 AC 720

13 5.5 62.4 AC 720

16 5 68.7 AC 720

17 5.4 65.8 AC 720

18 5.9 76.5 AC 720

20 6 70.4 AC 720

22 6.2 76.7 AZ 720

28 6.2 69.5 AZ 720

29 6.1 76.1 AZ 720

31 6.1 69.7 AZ 720

33 5.2 75.5 AZ 720

35 5.6 69.5 AZ 720

37 5 61.9 AZ 720

38 5.8 86.3 AZ 720

47 5.1 75.4 AZ 720

48 5.8 58.4 AZ 720

50 5.7 61.1 B 720

51 5.3 51.9 B 720

53 5.3 67.6 B 720

55 5.4 57.2 B 720

60 5.4 93.1 B 720

62 5.2 60.7 B 720

65 6.3 73.3 B 720

73 7 81 B 720

75 6.1 25.2 B 720

77 5.7 79.4 B 720

78 6.2 45.5 G 720

79 5.6 31.5 G 720

83 4.7 . G 720

90 5.7 . G 720

95 5.9 . G 720

98 5 . G 720

106 6.4 36 G 720

115 6.1 . G 720

118 5.9 . G 720

123 5 56.7 G 720

125 6 89.8 H 720

126 5.7 57.8 H 720

129 5.3 59 H 720

133 6 53.9 H 720

134 5.8 68.3 H 720

139 5.1 60.5 H 720

147 6.3 62.9 H 720

152 6.1 55.5 H 720

153 5 51.1 H 720

155 5.7 56.8 H 720

158 5.4 49.8 J 720

162 5.9 59.3 J 720

163 5 60.5 J 720

165 5.7 56.6 J 720

166 6.4 55.5 J 720

168 6.1 69 J 720

173 5.6 54.9 J 720

176 5.6 55.7 J 720

177 6.2 64.7 J 720

180 5.2 49 J 720

;

Proc mixed data=one method=type3;

Class TRT Block;

Model FBW=TRT IBW/s;

random Block;

Lsmeans TRT/PDIFF ADJUST=dunnett;

run;

Quit;

Cotton - Initial

dm 'log;clear;output;clear';

options nodate nocenter pageno=1 ls=78 ps=55;

title1 "CHeck Data";

data one;

input InsectID $ IBW FBW TRT $ block $;

datalines;

5 6.8 29.5 AC 720

6 7.6 23.1 AC 720

11 7.2 33.7 AC 720

12 7.7 40.2 AC 720

23 6.8 40 AC 720

24 7.9 23.2 AC 720

25 7.1 23.7 AC 720

27 8.1 31.8 AC 720

32 6.7 26 AC 720

36 7.2 32.6 AC 720

41 8.2 39.6 G 720

42 8.2 34.6 G 720

43 8.1 32.4 G 720

49 8 32.1 G 720

54 8 34 G 720

56 6.9 23.6 G 720

57 7.5 36.8 G 720

63 7.5 30.1 G 720

68 7.2 23.1 G 720

70 7.1 32.3 G 720

71 7.3 33.8 B 720

73 7 26.5 B 720

74 7.9 30.8 B 720

76 8.2 32.7 B 720

81 7.7 31.1 B 720

82 6.8 36.4 B 720

85 7.6 33.9 B 720

88 7.3 37 B 720

89 7 42.4 B 720

93 6.6 40 B 720

96 6.9 32.6 AZ 720

97 7.8 50.2 AZ 720

104 6.7 28.5 AZ 720

105 8.2 41.9 AZ 720

107 7.6 30.7 AZ 720

108 7.9 34.4 AZ 720

110 7 36.9 AZ 720

111 7 32.2 AZ 720

112 7.5 37 AZ 720

113 6.7 31.4 AZ 720

116 7.9 35.3 H 720

119 7.6 36.6 H 720

130 7.2 38.2 H 720

131 7.3 32 H 720

132 7.8 24.3 H 720

136 7.6 38.3 H 720

140 7.6 33.3 H 720

144 7.3 35.5 H 720

145 7.4 28.6 H 720

146 8.1 29.9 H 720

149 7.3 7.4 J 720

154 7 . J 720

156 8.2 11.1 J 720

157 6.5 . J 720

161 6.7 . J 720

164 7.7 12.7 J 720

171 7.7 23.8 J 720

172 7.3 12.8 J 720

174 7.9 23.1 J 720

175 7.3 26.4 J 720

1 5.5 37.7 AC 713

7 6.6 35.9 AC 713

9 5.4 40 AC 713

13 6.6 27.7 AC 713

17 6.3 32.5 AC 713

22 6.6 48.6 AC 713

27 6.5 31.6 AC 713

33 5.5 26.6 AC 713

43 5.5 29.1 AC 713

45 6.5 34.3 AC 713

51 6.6 35.1 G 713

53 5.9 23 G 713

60 6.7 62.7 G 713

61 5.9 48.5 G 713

62 5.7 29.8 G 713

65 5.6 29.5 G 713

72 5.8 24.6 G 713

77 5.5 23.9 G 713

86 5.8 57.7 G 713

94 6.1 62.5 G 713

105 6.8 52.1 B 713

106 6.7 . B 713

107 6.5 53 B 713

110 6.2 62.6 B 713

111 5.6 28.9 B 713

113 5.7 36.8 B 713

118 5.8 63.5 B 713

126 6.1 36.2 B 713

135 5.5 59.9 B 713

136 7 69.1 B 713

140 5.5 76.8 H 713

141 6.9 84.8 H 713

148 6.6 37 H 713

149 5.9 31.7 H 713

157 5.9 44.4 H 713

159 6.9 . H 713

163 6.1 60.8 H 713

165 6.6 44.8 H 713

169 6.5 39.2 H 713

177 6.4 44.2 H 713

179 6 23.5 J 713

188 5.4 14.7 J 713

189 5.4 16.6 J 713

208 6.2 17.9 J 713

212 5.8 17.9 J 713

220 5.9 26 J 713

222 6.4 17.8 J 713

232 5.6 15.5 J 713

234 5.9 15.9 J 713

235 5.4 19.6 J 713

236 6.7 37.6 AZ 713

238 6.6 66.2 AZ 713

239 5.8 44.6 AZ 713

244 5.6 26.4 AZ 713

246 6.6 33.7 AZ 713

247 5.5 48.4 AZ 713

251 6.3 35.7 AZ 713

254 5.8 30 AZ 713

255 6 32.9 AZ 713

256 5.4 37.5 AZ 713

2 5.1 11.2 AC 803

3 9.7 26 AC 803

4 6.2 . AC 803

5 7.7 14.5 AC 803

7 6.5 21.8 AC 803

8 7.6 15.3 AC 803

9 5.5 17.6 AC 803

10 5.6 13.3 AC 803

12 7.7 24 AC 803

15 6.7 19.3 AC 803

17 6 28 AZ 803

22 8.2 29.8 AZ 803

24 7.5 26.7 AZ 803

25 7.9 29.3 AZ 803

27 8 30.7 AZ 803

29 9.9 37.4 AZ 803

30 6.4 24.5 AZ 803

31 9.1 33.4 AZ 803

33 8.4 30.9 AZ 803

34 9 19 AZ 803

35 6.4 11.3 B 803

36 6.8 28.2 B 803

41 6.4 21.2 B 803

42 6.3 11.7 B 803

43 7.4 29.2 B 803

44 5.5 24.4 B 803

46 5.8 17.1 B 803

47 9.7 21.3 B 803

50 7.7 14.8 B 803

53 8.9 32.8 B 803

54 7.2 10.9 G 803

55 7.2 13.7 G 803

56 7.1 11.5 G 803

57 8.2 28.2 G 803

59 7.4 22.6 G 803

58 11.9 38.9 G 803

62 6.5 10.6 G 803

63 8.5 20.3 G 803

67 8.8 34.6 G 803

68 6.8 19 G 803

69 8.2 22.6 H 803

70 9.1 26.6 H 803

72 9.8 31.5 H 803

73 8.9 32.1 H 803

75 5.8 22.8 H 803

76 7.3 22.4 H 803

77 6.2 13.2 H 803

80 5.4 23.3 H 803

81 9.8 27.3 H 803

82 9.7 28.5 H 803

83 7.8 12.8 J 803

84 5.7 8.2 J 803

86 8.1 12.1 J 803

87 9.6 13 J 803

89 6.1 . J 803

90 7.9 . J 803

91 6.1 9.8 J 803

93 6.6 8.2 J 803

94 6.8 . J 803

95 7.2 . J 803

;

Proc mixed data=one method=type3;

Class TRT Block;

Model FBW=TRT IBW/s;

random Block;

Lsmeans TRT/PDIFF ADJUST=dunnett;

run;

Quit;

Soybean – Initial

dm 'log;clear;output;clear';

options nodate nocenter pageno=1 ls=78 ps=55;

title1 "CHeck all soybean Data";

data one;

input InsectID $ IBW FBW TRT $ Block $;

datalines;

98 7.2 29.5 AC 127

120 7.2 28.7 AC 127

122 7.4 29.2 AC 127

83 7.8 37 AC 127

97 7.9 41.5 AC 127

115 8.1 44.3 AC 127

119 8.2 42 AC 127

87 8.3 27.8 AC 127

108 8.3 21.3 AC 127

117 8.3 49.5 AC 127

77 7.1 24.1 AZ 127

211 7.1 29.4 AZ 127

51 7.3 52.6 AZ 127

205 7.4 48.8 AZ 127

80 7.7 15 AZ 127

81 7.8 37 AZ 127

202 8 44.9 AZ 127

75 8.1 25.2 AZ 127

79 8.1 49.3 AZ 127

201 8.4 55.7 AZ 127

230 7.3 26 B 127

237 7.3 38.3 B 127

243 7.3 42.9 B 127

217 7.7 26 B 127

232 8.1 12 B 127

222 8.3 26.3 B 127

235 8.3 50.1 B 127

236 8.4 33.1 B 127

215 8.5 53.4 B 127

227 8.5 37.3 B 127

24 7.1 19.7 G 127

32 7.4 30.7 G 127

33 7.4 15.9 G 127

14 7.5 16 G 127

12 7.6 37.8 G 127

17 7.6 33.2 G 127

58 8.1 52 G 127

47 8.3 23.3 G 127

25 8.4 33.5 G 127

160 7 41.3 H 127

164 7.1 21.1 H 127

194 7.3 51.7 H 127

171 7.4 39.9 H 127

198 7.5 61.9 H 127

163 7.6 26.7 H 127

184 7.6 58.8 H 127

177 7.7 29.1 H 127

182 8 28.2 H 127

193 8.5 69.7 H 127

153 7 21.9 J 127

121 7.4 25.6 J 127

143 7.4 18.6 J 127

125 7.6 25.7 J 127

144 7.6 24.1 J 127

150 7.6 19.9 J 127

141 7.8 21.6 J 127

148 7.9 18.5 J 127

155 8 30.1 J 127

151 8.4 20 J 127

9 4 68.1 AC 518

13 4 53.3 AC 518

2 4.3 26.9 AC 518

7 4.3 65.3 AC 518

15 4.3 71.8 AC 518

11 4.6 71.5 AC 518

1 4.9 . AC 518

6 4.9 78.9 AC 518

4 5.2 88.1 AC 518

3 5.3 80 AC 518

23 4.3 69.7 AZ 518

35 4.4 61.2 AZ 518

17 4.5 77.7 AZ 518

21 4.5 96.2 AZ 518

24 4.8 60.5 AZ 518

34 5 57.3 AZ 518

16 5.2 91.4 AZ 518

33 5.8 81.6 AZ 518

20 6.2 108.4 AZ 518

28 6.5 77.4 AZ 518

42 4 49 B 518

38 4.5 55.7 B 518

37 4.6 77 B 518

50 4.7 46.5 B 518

44 4.8 68.9 B 518

36 5.1 61.3 B 518

39 5.4 78.3 B 518

45 5.9 88.7 B 518

43 6.6 104.9 B 518

49 6.6 102.8 B 518

58 4.2 47.9 G 518

64 4.3 65.1 G 518

65 4.5 77.3 G 518

55 4.8 29.3 G 518

67 5.1 86.2 G 518

56 5.2 75.1 G 518

63 6.3 59.2 G 518

61 6.5 86.5 G 518

66 6.7 96 G 518

59 6.8 76.4 G 518

74 4.7 73.3 H 518

75 4.7 60.3 H 518

77 5 81.2 H 518

69 5.1 80.5 H 518

72 5.2 85.6 H 518

81 5.4 . H 518

70 5.7 99.5 H 518

68 6 113.7 H 518

80 6.4 109.1 H 518

78 6.5 90.6 H 518

94 4.4 32.7 J 518

82 4.6 34.2 J 518

90 4.9 52.3 J 518

93 4.9 47.9 J 518

91 5.2 56.6 J 518

96 5.4 49.3 J 518

88 5.9 36.7 J 518

92 5.9 61 J 518

95 6.2 49.7 J 518

87 6.5 50.8 J 518

4 3 8.5 AC 727

12 3 14.9 AC 727

13 3.3 14.2 AC 727

5 3.8 13.1 AC 727

7 3.8 16 AC 727

6 4 16.9 AC 727

8 4.1 14 AC 727

11 4.1 15 AC 727

3 4.5 13.1 AC 727

10 4.5 18.5 AC 727

30 3 12.8 AZ 727

32 3.2 12.6 AZ 727

21 3.9 14.2 AZ 727

22 3.9 11.8 AZ 727

31 3.9 14.9 AZ 727

24 4 12.1 AZ 727

15 4.1 17.9 AZ 727

26 4.1 18.5 AZ 727

18 4.6 19.7 AZ 727

25 5.1 15.2 AZ 727

38 3 13.9 B 727

42 3.2 13.1 B 727

43 3.2 15.1 B 727

40 3.3 25.3 B 727

45 3.3 13.5 B 727

37 3.9 14.1 B 727

44 4.1 15.3 B 727

36 4.2 17.6 B 727

41 4.8 22.8 B 727

34 5.1 18.2 B 727

68 3 . G 727

51 3.1 12.2 G 727

70 3.4 14.2 G 727

49 3.7 13.1 G 727

66 4.1 15.9 G 727

71 4.1 15.3 G 727

64 4.2 14.6 G 727

69 4.3 17.3 G 727

52 4.4 19.2 G 727

67 5.2 23.1 G 727

11 8.5 29.6 G 727

78 3.1 12.6 H 727

82 3.3 15.3 H 727

74 3.4 11 H 727

76 3.4 13.2 H 727

73 3.6 13.3 H 727

79 3.6 13.4 H 727

81 3.6 17.5 H 727

83 3.9 15 H 727

72 4 18.8 H 727

75 4.4 15 H 727

90 3 8.5 J 727

87 3.1 6.1 J 727

96 3.5 12.5 J 727

95 3.6 7.7 J 727

84 4 9.4 J 727

92 4.1 10.5 J 727

89 4.2 10.4 J 727

94 4.3 10.3 J 727

91 4.5 12.5 J 727

85 4.8 12.6 J 727

;

Proc mixed data=one method=type3;

Class TRT Block;

Model FBW=TRT IBW/s;

random Block;

Lsmeans TRT/PDIFF ADJUST=dunnett;

run;

Quit;

Rice – initial

dm 'log;clear;output;clear';

options nodate nocenter pageno=1 ls=78 ps=55;

title1 "Rice Combined";

data one;

input InsectID $ IBW FBW TRT $ Block $;

datalines;

106 3.8 22.8 J 127

15 6.5 30.8 J 127

21 6.8 34.3 J 127

172 3.4 9.8 J 127

28 6.3 16.9 J 127

234 3.4 9.2 J 127

30 6.7 29.8 J 127

31 5.6 22.2 J 127

55 5.4 21 J 127

10 10.5 73.9 J 127

64 6.4 38.1 B 127

69 6.6 38 B 127

70 6.7 39.6 B 127

71 10.7 50.3 B 127

86 6.9 31.4 B 127

93 6.2 59.9 B 127

100 5.4 34.6 B 127

102 6.7 40.6 B 127

103 6.2 29 B 127

104 5.5 26.4 B 127

107 5.6 29.9 AZ 127

111 6.8 57.1 AZ 127

114 5.9 23.7 AZ 127

124 4.6 24.8 AZ 127

126 6.6 48 AZ 127

133 6.5 32.1 AZ 127

134 4.5 . AZ 127

136 4.7 19.6 AZ 127

131 10.9 79.6 AZ 127

139 4.6 36.1 AZ 127

145 11.1 74.5 G 127

142 5.8 29.5 G 127

147 4 18.6 G 127

159 5.6 33.2 G 127

161 4.9 26.2 G 127

162 6.2 22.8 G 127

165 6.8 36.1 G 127

168 6.3 48.2 G 127

170 5.3 24.8 G 127

174 6.4 28.2 G 127

176 5.3 11.7 H 127

178 6.5 36.5 H 127

183 6.3 29.3 H 127

186 5.7 48.9 H 127

187 10.7 75.3 H 127

189 6.8 35.9 H 127

190 6.8 40.9 H 127

192 10.6 89.3 H 127

197 4.7 37.9 H 127

204 6.6 65.7 H 127

212 6.4 33.1 AC 127

213 5.9 33.9 AC 127

223 10.5 73.1 AC 127

226 6.8 36.7 AC 127

229 5.1 54.2 AC 127

242 4.9 21.4 AC 127

245 6.4 26.9 AC 127

246 5.8 38.2 AC 127

247 6.1 46.3 AC 127

248 5.4 103.3 AC 127

4 4.5 48.9 AZ 68

8 7.7 80.1 AZ 68

9 5 67.5 AZ 68

10 5 62.2 AZ 68

11 5.9 54.3 AZ 68

12 4.2 43.6 AZ 68

16 5.4 53.5 AZ 68

17 7.4 78.3 AZ 68

19 4.4 50.5 AZ 68

20 7.4 97.3 B 68

22 4.9 25.4 B 68

23 4 49.3 B 68

29 5.7 71.5 B 68

32 4.3 53 B 68

33 6 61.3 B 68

35 4.5 73.8 B 68

36 4.4 38.2 B 68

37 5.4 52.6 B 68

38 4.6 44.6 B 68

39 4 47.6 G 68

40 4.9 55.6 G 68

41 5 40.9 G 68

42 5.7 63.7 G 68

43 6.5 65.3 G 68

44 3.7 33.4 G 68

45 7.4 60 G 68

48 4.9 45.6 G 68

50 7.2 107.7 G 68

49 8.3 89.5 G 68

56 7.1 121.1 H 68

57 5.1 72.7 H 68

59 3.4 21.6 H 68

64 5.2 54.8 H 68

68 3.6 25.4 H 68

75 4 50.3 H 68

78 5.4 42.2 H 68

79 4.3 50.5 H 68

83 7.7 107.9 H 68

87 4.5 59.6 H 68

93 6.3 93.6 J 68

94 5.1 62.1 J 68

95 4.8 55.5 J 68

96 6.9 60.9 J 68

97 6.4 59.2 J 68

98 6.5 59.5 J 68

99 5.4 63.2 J 68

100 4.9 59.3 J 68

101 6.5 . J 68

102 5.2 58.9 J 68

103 6.6 78.4 AC 68

105 4.4 56.1 AC 68

106 7.4 70.6 AC 68

107 5.4 50.5 AC 68

112 6.1 60.3 AC 68

118 3.7 45.2 AC 68

119 7.6 81.4 AC 68

120 4.8 57.2 AC 68

122 4.2 42.1 AC 68

124 3.9 52.6 AC 68

6 3.3 57.3 AC 713

20 3.6 18.9 AC 713

21 3.7 37.7 AC 713

25 3.7 46.9 AC 713

32 3.7 61.7 AC 713

35 4.2 66.5 AC 713

38 3.8 56 AC 713

39 3.8 . AC 713

40 3.7 63.4 AC 713

41 3.5 38.3 AC 713

44 3.6 45.9 J 713

46 3.8 49.4 J 713

48 3.8 49 J 713

49 4.1 54.4 J 713

50 3.9 53.7 J 713

56 4.1 45.7 J 713

68 4.2 59.1 J 713

70 3.3 56.4 J 713

73 3.6 28.4 J 713

76 4.1 66.8 J 713

78 3.8 45.2 G 713

83 3.8 50.1 G 713

91 4.1 69 G 713

92 3.8 56.6 G 713

93 4 50.7 G 713

102 3.3 51.4 G 713

109 3.5 62.4 G 713

115 3.9 46.7 G 713

117 4.1 88.4 G 713

124 3.3 38.4 G 713

128 4.1 52.8 AZ 713

133 4 69.3 AZ 713

143 4.1 72.3 AZ 713

145 3.3 48.8 AZ 713

152 3.6 34.1 AZ 713

155 3.3 46.8 AZ 713

168 3.7 44.5 AZ 713

172 3.1 41.6 AZ 713

173 3.6 34.8 AZ 713

174 4.1 72.3 AZ 713

176 4 72.2 H 713

180 3.4 64.1 H 713

184 3.1 55 H 713

185 3.4 58.2 H 713

187 3.8 50.4 H 713

190 4.1 67.6 H 713

195 4 46.9 H 713

197 3.7 54.5 H 713

199 3.6 44.5 H 713

202 4 51.8 H 713

204 3.1 50.5 B 713

205 3.9 60.6 B 713

214 3.1 43.5 B 713

215 3.3 56.3 B 713

216 3.8 48.1 B 713

219 4 48.1 B 713

226 3.5 35.6 B 713

242 3.8 49.1 B 713

245 3.9 46 B 713

248 3.2 37.9 B 713

;

Proc mixed data=one method=type3;

Class TRT Block;

Model FBW=TRT IBW/s;

random Block;

Lsmeans TRT/PDIFF ADJUST=dunnett;

run;

Quit;

Corn Surfactant

dm 'log;clear;output;clear';

options nodate nocenter pageno=1 ls=78 ps=55;

title1 "John Research";

data one;

input InsectID $ IBW FBW TRT $ EXP $;

datalines;

65 11.2 149.1 t 116

66 10 124.9 t 116

67 8.8 145.6 t 116

68 12.3 167.3 t 116

69 8.7 129.3 t 116

70 8.2 139.1 t 116

71 12.6 163.4 t 116

72 9.7 125.9 t 116

73 8.4 107.7 t 116

75 11.8 99.5 t 116

76 7.4 95 x 116

77 7.8 111 x 116

78 8.5 124.9 x 116

79 9.1 139.5 x 116

80 9.8 111.5 x 116

81 8.5 140.4 x 116

82 9.3 142.3 x 116

83 9.7 136.3 x 116

84 12 127.3 x 116

85 12.9 136.5 x 116

86 8.9 148.1 p 116

87 11.6 166.5 p 116

88 11.7 158.8 p 116

89 8.8 119.3 p 116

90 10.3 149.3 p 116

91 12.6 141.1 p 116

92 8.2 114.6 p 116

93 11.6 149.6 p 116

94 10.5 48.9 p 116

95 9.2 89 p 116

98 15.9 149.7 2j 116

99 10.4 149.9 2j 116

101 9.1 155.4 2j 116

102 9.2 63.3 2j 116

103 8.1 138.1 2j 116

104 11.2 150.8 2j 116

105 8 136.4 2j 116

106 11.7 165.1 2j 116

110 8.8 157.5 2j 116

112 11.7 160.2 2j 116

113 9.4 116.4 5x 116

115 7.6 86.6 5x 116

117 7.1 119.7 5x 116

118 12.1 136.1 5x 116

119 12.9 146.8 5x 116

123 8.7 131.6 5x 116

125 7.5 134.2 5x 116

127 9 108.1 5x 116

130 10.6 134.1 5x 116

131 9.4 127.9 5x 116

132 8 82.6 5d 116

133 9.4 . 5d 116

135 10.1 142.6 5d 116

136 9.8 136.6 5d 116

137 9.9 152.3 5d 116

138 10.7 130.7 5d 116

139 8.4 125.2 5d 116

140 10.2 148.7 5d 116

141 9.1 142.3 5d 116

143 9 81.5 5d 116

145 9.2 134 5j 116

149 8.8 140.7 5j 116

150 10.7 130.5 5j 116

151 8.6 128.2 5j 116

152 8.1 124.9 5j 116

153 9.9 71.3 5j 116

154 11.3 140.5 5j 116

156 11.4 145.5 5j 116

157 7.9 81.3 5j 116

158 13.2 158.9 5j 116

162 12.3 157.7 c 116

164 8.2 137.3 c 116

165 8.7 144.8 c 116

167 7.6 . c 116

168 8.2 139.5 c 116

170 9.1 101.2 c 116

171 10.5 141.3 c 116

172 8.6 143.7 c 116

173 9.4 149.9 c 116

174 8.3 134.7 c 116

176 8.1 130.3 d 116

178 8.8 88.1 d 116

179 9.6 138 d 116

181 7.2 122.4 d 116

182 7.9 105.9 d 116

183 12.2 172.4 d 116

184 12.8 160.6 d 116

185 9.8 87.8 d 116

187 7.5 128.2 d 116

189 8.6 147.4 d 116

191 9 129.9 5t 116

192 8.4 130.8 5t 116

194 11.1 91.5 5t 116

195 10.9 111.3 5t 116

196 7 76.1 5t 116

197 7.8 109.5 5t 116

201 7.3 64.5 5t 116

202 8 104.5 5t 116

203 7.8 109.8 5t 116

205 9.8 144.2 5t 116

207 7.7 98.3 5p 116

209 13.2 128 5p 116

210 11.9 130.2 5p 116

211 9.4 120.6 5p 116

212 8.7 112.6 5p 116

213 8.9 89.4 5p 116

218 13.3 110.5 5p 116

220 8.8 81.4 5p 116

223 11.5 85.6 5p 116

224 8.5 114.6 5p 116

1 10.6 113.3 t 928

2 11.1 71.7 t 928

3 8.8 61.7 t 928

4 9 107.8 t 928

6 13.3 142.3 t 928

7 8.2 34.2 t 928

8 6.1 . t 928

10 11.5 136.4 t 928

11 9.7 108.1 t 928

12 11.4 87.2 t 928

13 8.6 112.4 x 928

14 8.8 . x 928

15 5.3 51.6 x 928

16 11.4 114 x 928

19 4.9 . x 928

20 12.4 86.5 x 928

22 10.1 52.6 x 928

23 5.8 88.5 x 928

24 10.6 105.6 x 928

26 6.1 34.7 x 928

27 8.8 92.8 p 928

28 8.7 103.6 p 928

30 12.3 130.8 p 928

31 6.8 35.6 p 928

35 8.1 . p 928

36 12.5 . p 928

38 8.1 . p 928

39 10.7 . p 928

40 5.5 . p 928

42 11.3 . p 928

44 7.2 54.6 2j 928

45 6.3 65.1 2j 928

47 10 101 2j 928

48 9.4 . 2j 928

49 11.3 101.5 2j 928

50 9.9 117.7 2j 928

51 7.2 67.9 2j 928

52 6.8 33.2 2j 928

53 10 60.1 2j 928

55 5.9 45.7 2j 928

56 4.8 20.9 5x 928

59 13.3 70.9 5x 928

60 11.2 84.7 5x 928

61 9.8 69.3 5x 928

64 12.3 100.5 5x 928

65 4.6 61.7 5x 928

66 8.1 33 5x 928

67 6.9 46.3 5x 928

68 7.1 95.2 5x 928

69 7.5 62.6 5x 928

70 6.2 57.5 5d 928

72 6.5 20.5 5d 928

73 5.9 50.5 5d 928

74 6.5 18.2 5d 928

76 4.8 37.1 5d 928

77 6.5 32.9 5d 928

42 11.3 57.1 5d 928

43 13.5 81.6 5d 928

39 10.7 59.3 5d 928

83 4.9 73.9 5d 928

85 4.6 38.8 5j 928

87 3.6 28.2 5j 928

90 8.3 62.7 5j 928

91 10.8 64.8 5j 928

92 8.2 43.7 5j 928

93 10.4 97.9 5j 928

94 11.4 110.9 5j 928

95 6.1 . 5j 928

96 12.5 107.1 5j 928

98 8.1 102.6 5j 928

99 9.6 101.2 c 928

102 7.5 92.2 c 928

104 5.4 89.2 c 928

105 7.1 118.5 c 928

106 4.6 55.5 c 928

108 9.3 133.1 c 928

109 9.3 143.5 c 928

110 6.4 88.8 c 928

112 7.8 122.6 c 928

113 10.1 117.2 c 928

114 8.3 84.8 d 928

115 10.9 93.5 d 928

116 9.4 84.4 d 928

118 11.6 53.7 d 928

119 7.9 84.3 d 928

123 7.4 62.8 d 928

124 10 61.1 d 928

125 4.6 32.7 d 928

126 5.4 28.1 d 928

101 14.3 145.3 d 928

128 7.4 111.8 5t 928

129 5.4 . 5t 928

137 4.5 97.8 5t 928

133 5.1 31.6 5t 928

135 6.2 40 5t 928

136 6.7 49.3 5t 928

139 6.2 . 5t 928

140 5.3 62 5t 928

148 4.2 29.4 5t 928

143 7.9 38.6 5t 928

144 5.8 51.3 5p 928

145 9.7 64.8 5p 928

150 4.6 33.7 5p 928

151 9.3 86.5 5p 928

152 9.3 49.5 5p 928

156 10 63.4 5p 928

157 6.9 50 5p 928

158 8.2 58.9 5p 928

159 8.7 56 5p 928

160 13.1 90.1 5p 928

4 11.1 144.8 5t 1024

6 8.5 120.5 5t 1024

10 10.6 140.2 5t 1024

11 9.7 72.2 5t 1024

12 9.6 134.7 5t 1024

14 9.1 138 5t 1024

16 9.7 139.5 5t 1024

17 6.1 121.9 5t 1024

20 10.3 113.8 5t 1024

21 10.5 148.1 5t 1024

22 9.1 134.6 5x 1024

23 10.6 142.6 5x 1024

24 6.8 . 5x 1024

26 6.2 93.9 5x 1024

28 9.8 135.3 5x 1024

30 7.7 118.9 5x 1024

31 10.3 146.6 5x 1024

32 9 119.3 5x 1024

35 7.7 108.3 5x 1024

36 9.9 136.4 5x 1024

37 8.8 135.1 t 1024

38 7.7 81.5 t 1024

39 7.9 132.7 t 1024

40 7.9 138.3 t 1024

41 8.1 117.7 t 1024

42 8.4 121.2 t 1024

43 9.2 121.8 t 1024

44 10.2 153.9 t 1024

46 10 145.5 t 1024

47 9.4 109.4 t 1024

48 11.2 150.4 5d 1024

49 9.1 127.2 5d 1024

50 8.6 126.6 5d 1024

51 8 114.1 5d 1024

52 8.3 113.8 5d 1024

53 8.2 122.9 5d 1024

54 8.3 104.6 5d 1024

55 7.8 . 5d 1024

57 6.9 100.4 5d 1024

58 10.6 137.9 5d 1024

61 9.1 143.4 5p 1024

62 6.3 91 5p 1024

64 8.6 134.6 5p 1024

66 6.9 106.4 5p 1024

68 10.4 103.7 5p 1024

71 9.8 121 5p 1024

72 7.1 129.3 5p 1024

73 11.2 169.5 5p 1024

74 6.4 127.2 5p 1024

76 8.4 128.1 5p 1024

78 8.3 88.6 d 1024

79 10.3 148.9 d 1024

81 7 110.9 d 1024

82 7.3 127 d 1024

83 8.9 129.9 d 1024

84 8.8 126.2 d 1024

88 8.4 133.5 d 1024

89 8.6 90.9 d 1024

90 9.3 116.6 d 1024

91 8.9 128.8 d 1024

94 10.8 109.8 x 1024

96 10.4 146.4 x 1024

97 7.9 104.4 x 1024

98 6.5 100.3 x 1024

100 7.5 106.6 x 1024

102 8.8 110.3 x 1024

103 9.9 125 x 1024

104 7.7 100.7 x 1024

106 7.9 104.9 x 1024

107 7.9 112 x 1024

108 7.8 89.2 2j 1024

109 8.2 83.5 2j 1024

110 8.8 135.6 2j 1024

111 10.2 127.2 2j 1024

112 9.5 117.2 2j 1024

113 8.1 115.9 2j 1024

115 6.3 87.5 2j 1024

119 9.7 115.5 2j 1024

120 8 105.2 2j 1024

121 6.9 82.8 2j 1024

122 8.4 122.4 p 1024

126 7.9 107.2 p 1024

127 8.9 116.6 p 1024

128 8.4 120.9 p 1024

130 9.5 154.7 p 1024

131 9.8 137.7 p 1024

132 8.3 107.5 p 1024

135 9.9 129.3 p 1024

137 7.4 91.3 p 1024

138 9.4 119 p 1024

139 7.8 89.1 5j 1024

140 6.4 94.7 5j 1024

141 6.7 86 5j 1024

142 8.5 100.5 5j 1024

143 9.6 129.4 5j 1024

144 10.1 124.7 5j 1024

145 8.3 113.9 5j 1024

146 6.3 101.5 5j 1024

147 9.1 104.5 5j 1024

149 10.4 117.1 5j 1024

151 7.9 114.2 c 1024

152 10.3 143.5 c 1024

153 9.6 132.1 c 1024

154 10.2 118.3 c 1024

155 9.2 134.6 c 1024

156 8.9 138.2 c 1024

157 8.1 95.1 c 1024

158 8 121 c 1024

159 9.9 143.9 c 1024

160 8.5 134.2 c 1024;

Proc mixed data=one method=type3;

Class TRT;

Model FBW=TRT IBW/s;

Lsmeans TRT/PDIFF ADJUST=TUKEY;

ods output diffs=ppp lsmeans=mmm;

run;

%include 'E:pdmix800.sas';

%pdmix800(ppp,mmm,alpha=.05,sort=yes);

Run;

Quit;

Cotton Surfactant

dm 'log;clear;output;clear';

options nodate nocenter pageno=1 ls=78 ps=55;

title1 "John Research";

data one;

input InsectID $ IBW FBW TRT $;

datalines;

1 10.6 32.5 t 119

3 14.2 55.9 t 119

4 8.5 28.1 t 119

5 16.3 69.2 t 119

6 11 38.2 t 119

7 10.7 54.9 t 119

8 10 32.4 t 119

9 17.1 58.9 t 119

11 17.6 . t 119

14 10.5 33.5 t 119

16 12.5 52.8 x 119

17 12.9 61.1 x 119

18 12.6 26.2 x 119

19 12.7 30.4 x 119

20 12.9 31.5 x 119

22 8.8 27.2 x 119

23 7.6 34.2 x 119

24 6.2 16.5 x 119

25 11 . x 119

27 13.3 53.2 x 119

28 9.8 32.3 p 119

29 6.5 . p 119

30 13.7 80.9 p 119

31 9.8 32.8 p 119

33 12.2 43.2 p 119

34 14.1 63.3 p 119

36 17.5 97.1 p 119

37 15.9 72.6 p 119

39 15.6 90 p 119

40 15.6 67.6 p 119

43 11.1 34.9 2j 119

42 18.5 46.2 2j 119

45 17.2 55.1 2j 119

47 15.5 27.4 2j 119

48 12.3 26.3 2j 119

49 9.4 27 2j 119

50 6.3 12.4 2j 119

53 8.1 . 2j 119

54 6.5 14.4 2j 119

55 15.1 34.8 2j 119

56 10.8 28.3 5x 119

57 12.6 36.5 5x 119

58 8.5 30.3 5x 119

59 8.9 . 5x 119

60 14.6 56 5x 119

63 13.7 41.7 5x 119

68 18.6 35.9 5x 119

65 11 19.2 5x 119

66 14.7 41.4 5x 119

67 14.1 47.5 5x 119

69 9.2 27.8 5d 119

70 17 . 5d 119

71 9.6 29.8 5d 119

72 9.7 17.9 5d 119

74 8.8 29.5 5d 119

75 11.8 30.1 5d 119

76 9.3 16.3 5d 119

77 14.3 32.1 5d 119

78 15.9 39.6 5d 119

79 13.5 31.7 5d 119

80 12.7 31.8 5j 119

81 8 29 5j 119

82 11.3 28.3 5j 119

83 7.5 23 5j 119

84 6.9 10.5 5j 119

86 17.6 46.6 5j 119

89 9.3 24.1 5j 119

90 8.5 33.1 5j 119

91 9.6 24.2 5j 119

92 13.5 50.5 5j 119

95 8.4 27.1 c 119

96 14.2 59.2 c 119

97 9.3 32.1 c 119

98 9 35.9 c 119

99 11.1 28.1 c 119

100 7.5 28 c 119

105 9.5 28.8 c 119

106 8.4 27.2 c 119

107 9.9 34.1 c 119

108 6.7 23 c 119

111 6.5 . d 119

112 8.5 31.1 d 119

116 13.4 63.1 d 119

117 7.6 32.5 d 119

123 9 37.3 d 119

125 6.5 26.7 d 119

126 9.7 39.9 d 119

128 7.2 28.1 d 119

129 14.1 37.8 d 119

130 11.4 51.6 d 119

131 15.7 29.7 5t 119

133 12.4 30 5t 119

135 15.8 37 5t 119

136 13.9 46.7 5t 119

137 8.2 18.1 5t 119

138 13.6 41.4 5t 119

139 9.9 19.4 5t 119

141 14.2 27.6 5t 119

142 10.8 33.5 5t 119

134 18.6 39.4 5t 119

144 10.8 28.5 5p 119

145 12.7 35 5p 119

147 15.9 33.5 5p 119

149 11 25.9 5p 119

151 8.1 32.9 5p 119

152 15.4 59.9 5p 119

154 13 40.5 5p 119

155 7.5 27.1 5p 119

156 15.8 55.1 5p 119

157 16.1 40.2 5p 119

2 11 22.8 c 1012

4 10.2 29.9 c 1012

6 11.4 43.8 c 1012

7 10 30.9 c 1012

8 9.7 30.8 c 1012

11 11 22.8 c 1012

13 10.6 18.1 c 1012

14 6.6 . c 1012

15 7.8 20.1 c 1012

17 9.6 25 c 1012

21 9.1 27.3 2j 1012

22 5.3 12.4 2j 1012

23 9.4 17.8 2j 1012

24 9.1 19.4 2j 1012

25 5.5 . 2j 1012

26 10.5 15.1 2j 1012

27 10.6 20.8 2j 1012

28 8.3 15.9 2j 1012

30 8.9 18.1 2j 1012

31 10.9 32.2 2j 1012

19 12.8 44.4 5j 1012

34 11.4 28 5j 1012

35 7.7 17.9 5j 1012

36 9.5 23.2 5j 1012

37 7.5 21 5j 1012

38 9 21.3 5j 1012

39 7.3 23.4 5j 1012

42 6.3 20.2 5j 1012

43 7 17.5 5j 1012

44 8.1 25.8 5j 1012

45 8.4 21 d 1012

46 5.7 11.9 d 1012

48 9.9 28.7 d 1012

49 9.4 20.9 d 1012

50 8.3 15.5 d 1012

52 9.9 18.8 d 1012

53 8.2 24.1 d 1012

54 8.3 18.1 d 1012

55 7.2 18.9 d 1012

57 5.3 11.9 d 1012

58 8.4 20.3 5d 1012

60 10.7 31.8 5d 1012

61 7.6 18.1 5d 1012

62 8.4 15.7 5d 1012

64 5.5 . 5d 1012

65 7.5 9.4 5d 1012

66 9.5 . 5d 1012

67 6.6 . 5d 1012

68 7.8 23.2 5d 1012

69 8.4 . 5d 1012

72 9.3 26.6 p 1012

73 7.3 17.9 p 1012

74 6.6 29.6 p 1012

75 8.7 . p 1012

81 7.9 . p 1012

83 7.4 15.7 p 1012

86 7.3 15 p 1012

87 8.7 . p 1012

88 8.8 . p 1012

89 10.8 28 p 1012

90 12.2 32.2 5p 1012

93 6.4 . 5p 1012

94 7.3 18.5 5p 1012

96 6.7 22.1 5p 1012

100 10.4 23.1 5p 1012

101 9.4 26.9 5p 1012

102 6.7 13.8 5p 1012

103 10.6 34.5 5p 1012

105 7.9 30.9 5p 1012

107 7.3 23.4 5p 1012

108 6.3 . x 1012

109 6.9 24.5 x 1012

110 8.1 21.6 x 1012

111 5.4 20.3 x 1012

112 5.6 21.8 x 1012

113 6 29.1 x 1012

114 6.1 . x 1012

115 7.9 22.2 x 1012

116 9.2 35.9 x 1012

118 10.2 43.2 x 1012

119 7.7 25.8 5x 10^12^

120 8.4 28.9 5x 10^12^

121 8.5 34.7 5x 10^12^

122 9.4 37 5x 10^12^

123 9.1 29 5x 10^12^

124 11.4 37.6 5x 10^12^

125 10.6 42.8 5x 10^12^

127 6.2 14.6 5x 10^12^

128 6.1 23.9 5x 10^12^

129 7.9 27.7 5x 10^12^

131 5.7 18.5 t 1012

132 6.8 19.1 t 1012

133 6.8 25.7 t 1012

134 6.3 10.9 t 1012

136 5.3 . t 1012

137 5.4 18.3 t 1012

138 6.5 15.7 t 1012

139 9.4 37.5 t 1012

141 5.9 . t 1012

144 6.3 . t 1012

148 6.7 31.3 5t 1012

150 7 32 5t 1012

152 6.8 23.4 5t 1012

153 6.4 22.9 5t 1012

154 9.5 16.7 5t 1012

155 9.7 40.4 5t 1012

156 6.5 31.4 5t 1012

157 5.8 22.1 5t 1012

158 5.5 18.8 5t 1012

160 6.4 28.7 5t 1012

1 7.2 20.1 5d 97

3 7 41.1 5d 97

5 4.7 19.3 5d 97

6 5.1 17.9 5d 97

7 7.7 37.7 5d 97

8 7.1 41.7 5d 97

9 7.7 57 5d 97

10 5 19.7 5d 97

12 7.7 26 5d 97

13 5.4 35.3 5d 97

14 6.8 50.6 t 97

15 6.1 40.5 t 97

16 7.6 33.1 t 97

17 7.2 40.2 t 97

18 6 44.3 t 97

19 6.7 80.7 t 97

20 7.1 72.6 t 97

21 6.5 64.7 t 97

22 4.6 31.9 t 97

23 6.5 58.7 t 97

27 7.5 54.4 p 97

28 5.3 37.6 p 97

29 7.1 51.4 p 97

30 4.8 24.2 p 97

31 5.8 31.2 p 97

32 5.9 32.5 p 97

35 6.4 50 p 97

36 7.2 43.8 p 97

37 6 47.1 p 97

39 6.2 43.8 p 97

40 7.5 33.6 x 97

41 5.2 64.1 x 97

42 7.5 46.2 x 97

43 7.2 39 x 97

44 5.8 37.9 x 97

45 6 50.2 x 97

46 7 55.8 x 97

49 4.7 31.8 x 97

51 7.2 55.1 x 97

53 7.6 65.5 x 97

54 6.8 48.6 c 97

55 7.1 42.1 c 97

56 5.5 36.1 c 97

57 7 47.7 c 97

59 5.2 56.1 c 97

60 4.6 18.6 c 97

61 7.2 47.8 c 97

62 6.8 32.5 c 97

68 6 54.1 c 97

70 6.5 28.7 c 97

71 7.5 53.2 d 97

72 6.7 45.4 d 97

73 6.5 36.7 d 97

74 6.8 50.7 d 97

77 6.4 32.7 d 97

78 4.8 16.6 d 97

79 7.2 59.2 d 97

80 7 49.6 d 97

82 4.6 24.4 d 97

83 6.7 46.1 d 97

90 5.5 22.4 5j 97

91 6.8 39.1 5j 97

92 7 46.5 5j 97

93 5.8 39.1 5j 97

94 5 26.2 5j 97

96 5.6 41.9 5j 97

100 5.5 19.1 5j 97

101 4.6 29.1 5j 97

102 5.7 33.9 5j 97

103 6.5 39.2 5j 97

104 6.2 32.6 5x 97

105 4.6 21.6 5x 97

106 6.8 25.1 5x 97

107 6.5 13.4 5x 97

108 6.7 50.9 5x 97

109 5.5 36.4 5x 97

110 5.6 35.4 5x 97

112 5.6 25.5 5x 97

113 6.4 34.5 5x 97

114 5.1 23.2 5x 97

115 5.5 30.7 5p 97

116 5.1 24.5 5p 97

117 6 27.7 5p 97

118 6.2 35.1 5p 97

119 5.8 23.3 5p 97

122 5.6 21.9 5p 97

123 7.3 25.8 5p 97

126 6.9 37.2 5p 97

127 7.3 34.7 5p 97

128 7.1 37.5 5p 97

130 6.6 30.3 5t 97

132 4.9 22.6 5t 97

134 7.4 34.1 5t 97

135 4.9 28.3 5t 97

136 6 31 5t 97

137 6 27.3 5t 97

138 5.8 27.1 5t 97

140 5.6 16.2 5t 97

141 5.3 15.8 5t 97

144 6.2 32.9 5t 97

145 5.9 12.2 2j 97

146 5.3 15.5 2j 97

147 5.8 21.3 2j 97

148 5.6 29.2 2j 97

149 4.6 16.8 2j 97

150 7.5 22.1 2j 97

151 6.3 16.6 2j 97

154 7.3 30.2 2j 97

155 5.7 16.7 2j 97

157 7.3 17.7 2j 97

;

Proc mixed data=one method=type3;

Class TRT;

Model FBW=TRT IBW/s;

Lsmeans TRT/PDIFF ADJUST=TUKEY;

ods output diffs=ppp lsmeans=mmm;

run;

%include 'F:pdmix800.sas';

%pdmix800(ppp,mmm,alpha=.05,sort=yes);

Run;

Quit;

Soybean Looper – Experiment 3

dm 'log;clear;output;clear';

options nodate nocenter pageno=1 ls=78 ps=55;

title1 "SBLooperCombined";

data one;

input InsectID $ IBW FBW TRT $;

datalines;

32 103.3 236.2 c

34 74.6 206.9 c

35 83.2 228.2 c

36 93.1 197.1 c

38 105 252 c

43 92.3 206.1 c

44 106.7 237.1 c

45 90.3 226.8 c

47 102.4 222.8 c

48 101.3 227.9 c

50 95.3 196.1 c

52 90.4 257.3 c

53 99.9 240.6 c

54 98.5 239 c

56 76.8 229.4 j

59 104.6 258.9 j

60 101.8 249.5 j

64 96.8 277.8 j

65 90.1 216.5 j

67 88 221.3 j

68 92.8 193.3 j

72 71.9 190.1 j

73 74.3 165.7 j

75 73 196.5 j

80 83.6 236.2 j

85 71.3 211.7 j

86 95.9 194.3 j

87 78.8 163.4 j

91 81.4 223.5 j

95 82.4 180.5 j

3 14.6 106.9 c

4 15.4 155.8 c

5 14.9 119 c

7 12.2 99.9 c

8 15.7 123.1 c

9 14.8 149.2 c

11 13.4 129.7 c

13 15.3 149.6 c

15 12.5 136 c

19 15.2 119.5 c

20 13.1 144.8 c

21 14 114.1 c

22 17 152.5 c

25 13.6 142.4 c

26 11.1 109 c

47 12.4 106.3 j

48 16.4 119.4 j

51 16.9 77.5 j

52 12.6 94.9 j

53 12 87.9 j

54 13.6 106.4 j

55 14.1 93.8 j

56 15.3 77.3 j

58 15.6 81.7 j

59 14.4 137.1 j

60 12.4 82.9 j

61 14.3 109.9 j

62 14.4 83.4 j

63 16.2 87.2 j

64 15.8 116.1 j

2 98.7 210 c

3 94.2 192.8 c

5 91.1 197.8 c

7 100.7 207.6 c

8 99.3 211.5 c

10 103.5 212.9 c

11 90.3 195.3 c

12 103.2 201.5 c

14 103.1 225.3 c

15 89.4 202.4 c

16 94.9 203.3 c

17 89.9 202.6 c

18 93.9 207.5 c

21 85.1 201.9 c

41 101.6 193.3 j

42 89.3 183.5 j

43 95.8 167.8 j

44 95.2 204.4 j

45 95.3 177.9 j

46 97.7 220.9 j

48 98.4 224.2 j

51 96.6 180.7 j

52 86.4 217.2 j

55 101 206.8 j

62 94.1 186 j

63 98.1 156.3 j

64 98.1 181.6 j

58 85.2 182.8 j

;

title1 'Plot of the data with lines';

symbol1 v='j' i=rl c=black;

symbol2 v='c' i=rl c=red;

proc gplot data=one;

plot FBW*IBW=TRT;

run;

Proc mixed data=one method=type3;

Class TRT;

Model FBW=TRT IBW/s;

Lsmeans TRT/PDIFF;

ods output diffs=ppp lsmeans=mmm;

run;

Quit;

Fall Armyworm – Experiment 3

dm 'log;clear;output;clear';

options nodate nocenter pageno=1 ls=78 ps=55;

title1 "FAWCombined";

data one;

input InsectID $ IBW FBW TRT $;

datalines;

57 9.3 57.9 c

60 9 46.1 c

65 9 57.1 c

67 9.1 36.8 c

73 10.2 53.9 c

74 9 24.8 c

89 9.7 57.2 c

99 9.2 55.6 c

101 8.8 36.8 c

113 9.3 68.7 c

118 9.1 38.8 c

137 9.4 55.5 c

152 9.6 76.8 c

157 10 22.3 c

167 9.5 . j

169 9.6 29.2 j

173 9.6 38.2 j

179 10.3 25.2 j

199 8.9 29.7 j

206 9.9 33.8 j

208 10 30.9 j

210 9.4 . j

219 10.1 23.5 j

228 8.6 49.6 j

231 10.1 28.5 j

238 9.8 37.4 j

233 10.4 29 j

244 8.5 38 j

250 9.4 18.3 j

5 10.5 47.9 j

1 10 35.4 c

2 11.8 43.1 c

3 9.1 42.8 c

4 9.7 43.4 c

5 8.2 32.5 c

6 8.1 36.6 c

7 10.8 39.8 c

8 8.3 34.9 c

9 7.9 33.7 c

10 8.2 37.7 c

11 11.3 58.4 c

14 10.2 37.5 c

15 8.6 36.7 c

16 8.7 41.2 c

17 10.7 42.7 c

39 7.9 27.8 j

42 9.1 32.7 j

43 10.4 24.9 j

44 10.8 27.6 j

48 10.1 28.9 j

50 7.1 20.7 j

53 9.8 40.9 j

57 9.3 30.7 j

58 11.5 28 j

60 10.2 40.2 j

1 8.4 52.2 c

2 9.1 130.3 c

3 5.9 . c

4 7.4 59.9 c

6 8.2 79.9 c

7 6.8 49.2 c

8 7.4 69.1 c

9 8.8 82 c

10 7.8 62.5 c

11 8.9 60.3 c

13 6.9 51.5 c

15 5.9 78.9 c

16 9.9 92.5 c

18 10.3 87.7 c

19 10 103.9 c

47 7.6 24.6 j

48 7.7 44.1 j

49 6.5 24.5 j

51 10.5 36.9 j

53 6.6 24.6 j

54 9.3 35.2 j

55 8.7 26.1 j

57 6.6 30.1 j

58 9.7 33.3 j

59 9.5 37.9 j

60 9.6 32.8 j

61 6.3 30 j

62 11.1 46.8 j

63 10.9 36 j

64 7 48.5 j

;

title1 'Plot of the data with lines';

symbol1 v='j' i=rl c=black;

symbol2 v='c' i=rl c=red;

proc gplot data=one;

plot FBW*IBW=TRT;

run;

Proc mixed data=one method=type3;

Class TRT;

Model FBW=TRT IBW/s;

Lsmeans TRT/PDIFF;

ods output diffs=ppp lsmeans=mmm;

run;

Quit;
